# Synthetic heparan sulfate dodecasaccharides reveal single sulfation site interconverts CXCL8 and CXCL12 chemokine biology†
†Electronic supplementary information (ESI) available. See DOI: 10.1039/c5cc05222j



**DOI:** 10.1039/c5cc05222j

**Published:** 2015-08-03

**Authors:** Gordon C. Jayson, Steen U. Hansen, Gavin J. Miller, Claire L. Cole, Graham Rushton, Egle Avizienyte, John M. Gardiner

**Affiliations:** a Christie Hospital , Institute of Cancer Studies , University of Manchester , Manchester , M20 4BX , UK; b Manchester Institute of Biotechnology and School of Chemistry , University of Manchester , 131 Princess Street , M1 7DN , UK . Email: gardiner@manchester.ac.uk ; Tel: +44 (0)161 306 4530

## Abstract

The multigram-scale synthesis of a sulfation-site programmed heparin-like dodecasaccharide is described. Evaluation alongside dodecasaccharides lacking this single glucosamine O6-sulfation, or having per-O6-sulfation, shows that site-specific modification of the terminal glucosamine dramatically interconverts regulation of *in vitro* and *in vivo* biology mediated by the two important chemokines, CXCL12 (SDF1α) or CXCL8 (IL-8).

## Abstract 2

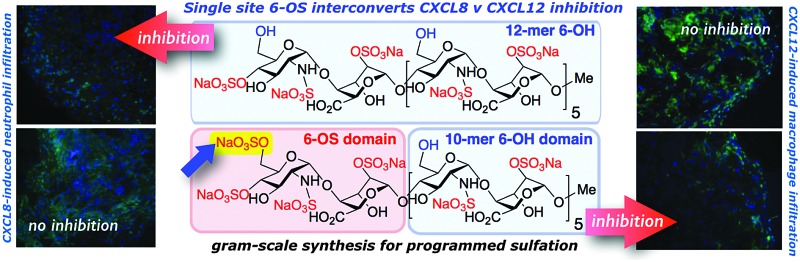
Multigram-scale synthesis of a sulfation-site programmed dodecasaccharide is described. CXCL8- and CXCL12-mediated *in vitro* and *in vivo* biology is shown to be regulated by a single sulfation site change.

The glycosylaminoglycan (GAG) heparan sulfate (HS) is a linear oligosaccharide polymer of alternating *N*-substituted glucosamine (GlcN) and a hexuronic acid, which is either glucuronic acid (GlcA) or the more conformationally-flexible iduronic acid (IdoA). Chains of approximately 50 and up to ∼200 monosaccharides are covalently linked to core proteins to form HS proteoglycans (HSPGs) that are embedded in the cell membrane or extracellular matrix, where they mediate interactions key to coordination of signaling, proliferation and stem-cell differentiation.^1^ HS binds over a hundred proteins^2^ and plays ubiquitous regulatory roles in angiogenesis, inflammation and stem-cell differentiation and thus HS-related oligosaccharides offer numerous potential applications as therapeutics.^3^ Following the discovery of the specific interaction between heparin and anti-thrombin III approximately thirty years ago and the major medical importance of heparin, there has been a continued search for H/HS-like structures with defined biological structural specificity for other targets. The success of Arixtra (Fondparinux) as a synthetic anti-coagulant LMWH heparin (5-mer) has illustrated the clinical potential for pure short synthetic heparin-related fragments.^4^ Much important biological regulation is effected by longer sequences and the great heterogeneity of natural extracts makes synthetic routes essential, however, particularly progression to *in vivo* biology of any homogenous longer heparin-like oligosaccharides has been precluded by lack of scalable syntheses. We recently exploited large-scale iduronate synthesis^5,6^ to generate gram-scale quantities of structurally-defined per-6-OH heparin-like dodecasaccharide **1**.^7^ This iterative synthetic strategy was also applied to synthesis of the new homogeneously per-6-*O*-sulfated heparin-like dodecamer **3**.^8^ We report here application to synthesis of the first example of a site-specifically mono-6-*O*-sulfated heparin-like dodecasaccharide, **2**. Provision of **2** on approximately 2 gram scale firmly establishes this approach as a practicable general route to various long heparin-like oligosaccharides on scales exceeding other GAG 12-mer syntheses by two orders of magnitude and underpins the practicability of delivering long synthetic GAGs for *in vivo* biology.

HS is organized into domains of high and low sulfation, with high sulfation domains consisting predominantly of GlcN- and O6, and IdoA O2 sulfation. These higher sulfation domains are principal effector parts of HS, binding ligand cytokines leading to receptor activation.^10,11^ Identifying new, discrete synthetic HS structures with defined biological effects is central to understanding GAG microstructure-specificity and the potential for discovery of new biomedical H/HS mimetics.^12^

GAGs are critically involved in chemokine biology, regulating oligomerization and phenotypic effects.^13,14^ HS-binding chemokines CXCL8 (IL-8), CCL19^15^ and CXCL12^16–18^ (SDF-1α) are involved in angiogenesis, metastasis and inflammation and are thus high value biomedical targets. Specific effector HS ligands for these chemokines remain undefined, and identification of specific structures which differentially modulate biological activity of these chemokines would provide new insight into GAG-chemokine chemical biology and potential for new drug developments.

Considerable evidence indicates that the overall level of glucosamine-6-*O*-sulfation is important for differential effects of H/HS oligosaccharides,^19^ and as a regulator of the biological activity of several HS-dependent cytokines, such as CXCL8^13,20^ and CXCL12^20,21^ as well as FGF2^22^ and VEGF.^14,21,23^ Prior work has indicated that homogenous sulfation levels and locations can affect the binding preferences of other GAGs to target proteins.^24,25^ Access to site-specific variations of longer HS sequences is essential to investigate whether site-specific 6-*O*-sulfation variations can determine biological effect in longer native-like ligands.

The syntheses of a series of three sulfation-programmed oligosaccharides (Fig. 1, **1–3**), and most particularly our capacity to deliver much larger scale syntheses than previously available, provides a tool-kit to interrogate the effects of both level and site-specificity of defined sulfation *in vitro* and *in vivo*. We report here that the presence or absence of a single site-specific sulfate interconverts CXCL8 or CXCL12 inhibitory effects between **1** and **2**, both *in vitro* and *in vivo*. These dramatic differential effects are not evident for the per-6-*O*-sulfated dodecasaccharide analogue **3**, therefore indicating here that charge density is not a critical mediator of cytokine-mediated effects, as previously suggested.^22^

**Fig. 1:**
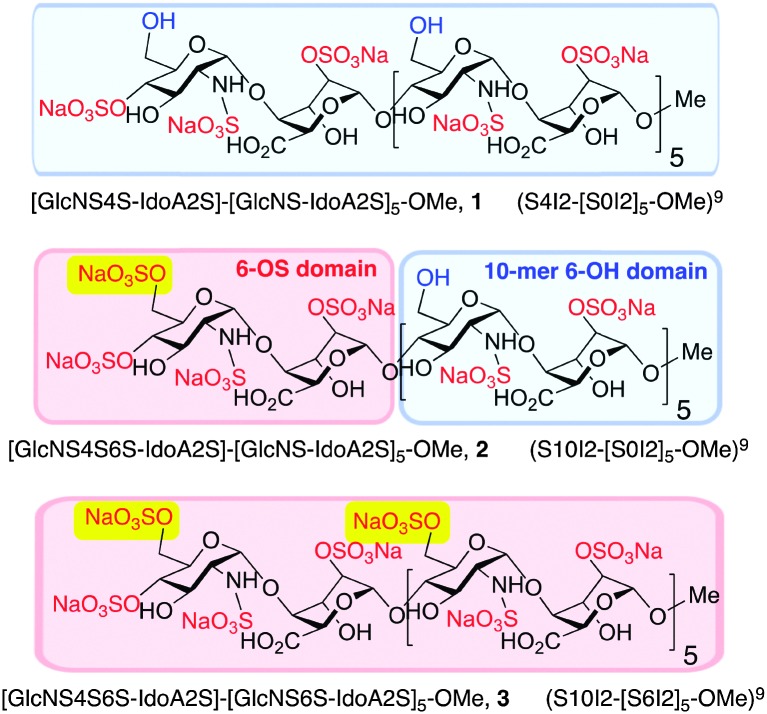
Site-specifically 6-*O*-sulfated dodecasaccharides: completely non-6-*O*-sulfated **1**,^5^ site-selectively mono-6-*O*-sulfated **2** and fully 6-*O*-sulfated **3**.^6^ [Blue shading: 6-OH microdomain; red-shading: 6-OS microdomain].

Generation of novel HS 12-mer **2** with an additional O6-sulfate at one specific terminus was achieved by capitalizing on the robust scalability demonstrated for prior synthesis of homogenous dodecasaccharides.^7^ Thus, 10-mer acceptor **4** was converted into the protected 12-mer **6** by coupling with O6-modified disaccharide donor **5**,^8^ which programmes for subsequent GlcN 6-*O*-sulfation at the non-reducing terminal unit only (Scheme 1), by tying the ultimate O6-sulfation fate to that of iduronate O2. The GlcN-IdoA disaccharide donor-based approach enabled this module switch to provide batch synthesis of protected 12-mer, on >5 gram scale. Saponification introduced free IdoA-2-OH groups and carboxylates and released the key single terminal GlcN 6-OH, generating partially protected 12-mer intermediate **6** on 3 g scale. *O*-Sulfation of **6** (yielding **7**) and subsequent debenzylation/azide reduction using Pd(OH)_2_/C generated ∼2 g of the site-specifically GlcN-6-*O*-sulfated penultimate dodecasaccharide **8**. Final *N*-sulfation afforded the novel target dodecasaccharide **2** on ∼2 g scale, demonstrating the generic scalability of this iterative synthetic approach.^7,8^

**Scheme 1:**
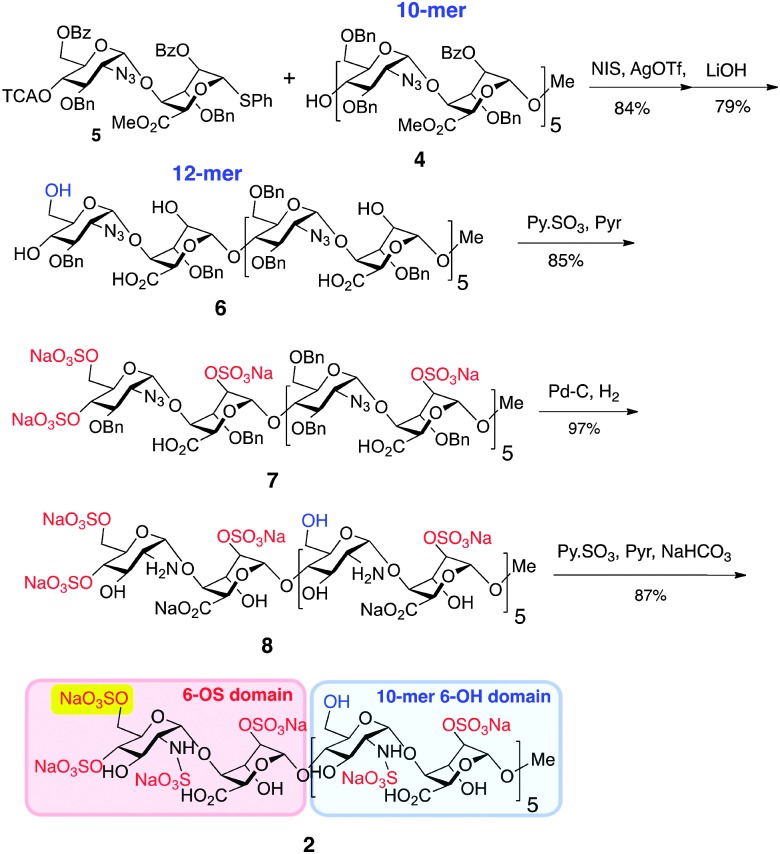
Synthesis of dodecasaccharide **2**.

The homogeneity of all materials was unambiguously confirmed by LCMS, MS and 800 MHz HSQC NMR analyses. Most particularly, ESI mass spectral data were fully consistent with a mono-O6-sulfated species, whilst the HSQC data provided confirmation of single site of sulfation significantly differentiated from the main-chain C6 methylene units (Fig. 2).

**Fig. 2:**
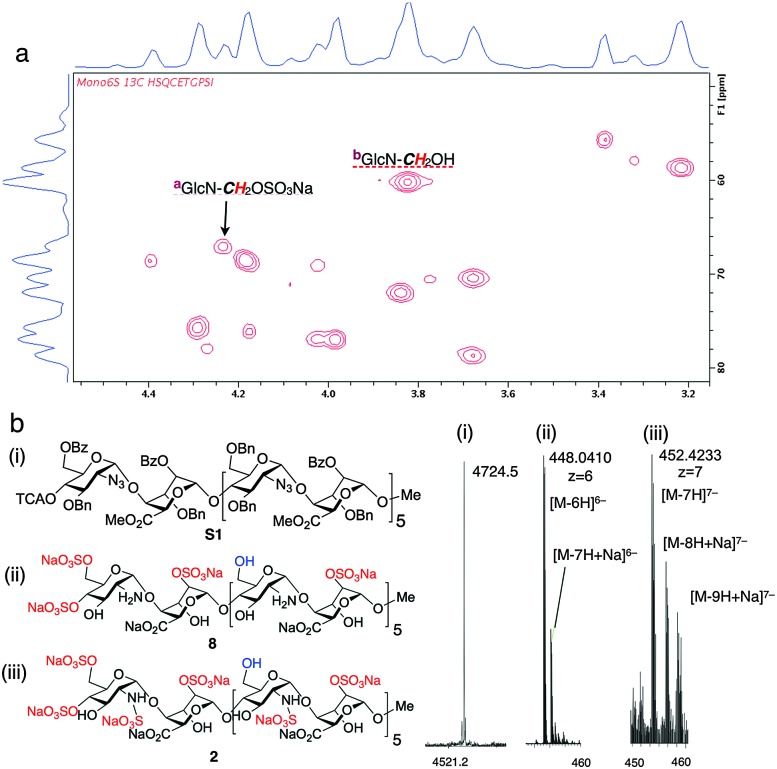
Site-specifically 6-*O*-sulfated dodecasaccharides: structural characterization. (a) 800 MHz HSQC NMR data confirming terminal ring 6-*O*-sulfation of dodecasaccharide **2** (HSQC overlay data for **2** and **1**; Fig. S22 in ESI,†). (b) (i) MALDI-TOF mass spectral base peak [M + Na]^+^ for the fully-protected species (ii) ESI mass spectral data for dodecasaccharide **8** (iii) ESI mass spectral data for site-specifically sulfated dodecasaccharide **2**.

This scalable access to the mono-6-*O*-sulfated dodecasaccharide **2** alongside fully 6-*O*-desulfated 12-mer, **1** ^7^ and the fully 6-*O*-sulfated 12-mer **3**,^8^ provided a toolkit to evaluate the biological effects of modifying a single sulfation site at the non-reducing terminal glucosamine in long synthetic heparin-like species, as well as control evaluation of full O6-sulfation both in a otherwise identical synthetic 12-mer (**3**) and a digest HS 12-mer. It must be emphasized that whilst all synthetic sequences shared the same terminal 4-*O*-sulfate, digest HS – the regular standard in biological assays – also lacks a 4-OH as digests contain a terminal unsaturation, and thus there is no rationale to consider the inclusion or absence of a 4-OH relevant, as this is invariably also a synthetic construct from other synthetic routes. Further, it is noted that the only variable between the synthetic species were 6-*O*-sulfation changes and any hypothetical relative effects from a 4-*O*-sulfate should therefore be discounted.‡
‡Migration and pERK signaling assays compared synthetic 4-OS-terminated per-6-OS sequence 3 with commercial digest-derived dp12 (ESI† Fig. S25) reinforcing that the 4-OS is not a significant variable. This is further supported by our migration and pERK signaling assays comparing synthetic 4-OS-terminated per-6-OS sequence **3** with commercial digest-derived dp12 (ESI,† Fig. S25). Therefore, the 4-OS is a constant and not an effector and this establishes that 4-OS terminated heparin-like oligosaccharides are bioactive *in vitro* and *in vivo* and thus also may adapt future approaches to synthesis of such oligosaccharides.

Multiple experimental models of cytokine-elicited endothelial cell biological responses provided clear, consistent evidence of an orthogonal impact on the biological effects of CXCL8 and CXCL12 by synthetic dodecasaccharides bearing either no sulfates on GlcN-O6 (**1**) or a single, non-reducing end GlcN-O6 sulfate (**2**). The fully 6-*O*-sulfated dodecasaccharide **3** did not show this inhibition switching, and its behaviour was very similar to that of de-polymerised digest dp12 derived from commercial heparin (ESI,† Fig. S25). This strongly reinforces evidence that the single site sulfation is the unique differential regulating this switch in chemokine effects and that that is indeed structure-specific.§
§Synthetic dodecasaccharides had no effect on the cells when they were not stimulated by cytokines or cells stimulated by non-HS dependent cytokines such as EGF and VEGF_121_.


Specifically, cytokine-induced endothelial cell monolayer wound healing models show strikingly different effects of dodecasaccharides **1–3** on CXCL8 and CXCL12-elicited endothelial migration (Fig. 3a and b, dotted boxes). Dodecasaccharide **1** was the most potent inhibitor of CXCL8-induced migration (Fig. 3a), but the weakest inhibitor of CXCL12-induced migration, whereas site-specifically sulfated **2** was the most potent inhibitor of CXCL12, whilst **1** showed very limited effect (Fig. 3b). Thus, addition of a 6-*O*-sulfate converts CXCL8-induced endothelial cell migration-inhibiting 12-mer **1** into a CXCL12-induced inhibitor **2**. The reversal of effects is highlighted by blue-dashed boxes in Fig. 3.

**Fig. 3:**
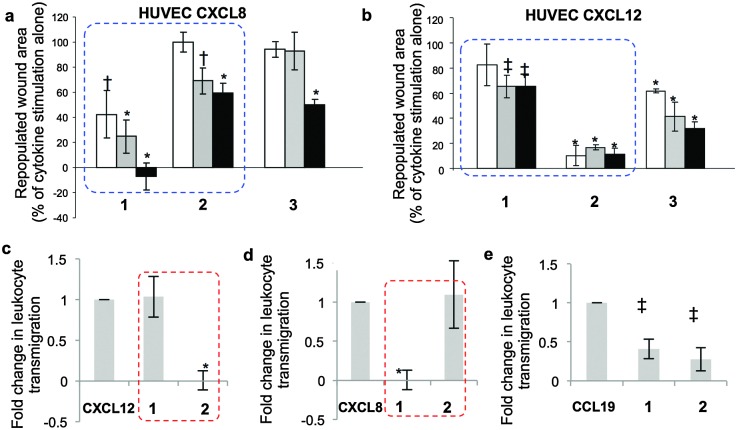
Differential effects of 12-mers **1–3** on CXCL8 and CXCL12-mediated functions*, in vitro*. (a and b) HUVEC migration in response to (a) CXCL8 and (b) CXCL12 ± 12-mers in wound-healing assay. [**1**, **2** and **3** at 1 μg ml^–1^ (white), 10 μg ml^–1^ (grey) or 50 μg ml^–1^ (black). Area repopulated in 24 h as percentage of the effect of cytokine without oligosaccharides (=100%)]. *, *p* < 0.001; †, *p* < 0.01; ‡, *p* < 0.05. (c–e) Inhibition of CXCL12, CXCL8 and CCL19-mediated leukocyte transmigration through an endothelial monolayer by **1** and **2**. Two experiments data as mean ± SD. * *p* < 0.01; ‡, *p* < 0.05. (c)–(e) Non-significant differential impacts of **1** and **2** on chemokine CCL19-mediated migration.

The effects on downstream signaling of the three dodecasaccharides on CXCL12-induced ERK1/2 phosphorylation and on CXCL8-induced STAT3 phosphorylation showed the same striking orthogonality of effects for **1** and **2**. The most potent inhibition of CXCL12 signaling (75% at 10 μg ml^–1^) was effected with site-specifically sulfated **2** while the most effective inhibitor of CXCL8 was **1**, reducing STAT3 phosphorylation >80% at 1 and 10 μg ml^–1^ (ESI,† Fig. S23).

To further validate the *in vitro* differential effects of the single sulfation-site programming and to show that this is specific for CXCL8–CXCL12, the effects of **1** and **2** on leukocyte transmigration through an endothelial monolayer were evaluated. Parallel experiments were undertaken using the homeostatic chemokine CCL19. Dramatic, near-orthogonal on-off relationships with respect to CXCL8 and CXCL12 were observed with **1** and **2** (Fig. 3c and d: red-dashed boxes illustrate the reversal of effects). In contrast, the impact of oligosaccharides on CCL19-mediated migration (Fig. 3e) showed similar and incomplete levels of inhibition with both **1** and **2**. These data demonstrate that regulation of the biology of specific chemokines can be mediated solely by the presence or absence of a single 6-*O*-sulfate moiety in a synthetic dodecasaccharide backbone, and regulate only specific chemokines.

Prior seminal work on *in vivo* biology of long H/HS has employed digest-derived heterogeneous HS^26^ and more recent studies also have evaluated non-GAG synthetic mimetics.^27,28^ Synthesis reported here of grams of structurally-defined oligosaccharides **1** and **2** enabled us to conduct the first *in vivo* studies with such oligosaccharides, in several biological models. Most importantly, was the objective of identifying whether the remarkable *in vitro* switch effects (*vide supra*) were observed *in vivo*. CXCL8 or CXCL12 and oligosaccharide-impregnated sponges were implanted subcutaneously in mice to determine the effects of the oligosaccharides on chemokine-induced biology (Fig. 4). CXCL8-mediated vascular density and neutrophil infiltration (Fig. 4a and b) were significantly inhibited by **1**, whereas site-specifically sulfated analogue **2** was ineffective. The converse situation was seen in inhibiting CXCL12-induced repopulation of sponges with host vasculature (Fig. 4c), infiltration of neutrophils (Fig. 4d) and macrophages (Fig. 4e, ESI,† Fig. 25a–e). These *in vivo* data show dramatically changed selectivity of the *in vivo* biological effects between the dodecasaccharides **1** and **2**, correlating with all the cell biology and signaling data. The only difference between **1** and **2** is the addition of a single site-specific GlcN6S in **2**. This is the first evidence for such defined degree of site-sulfation structural specificity modulating *in vivo* effects.

**Fig. 4:**
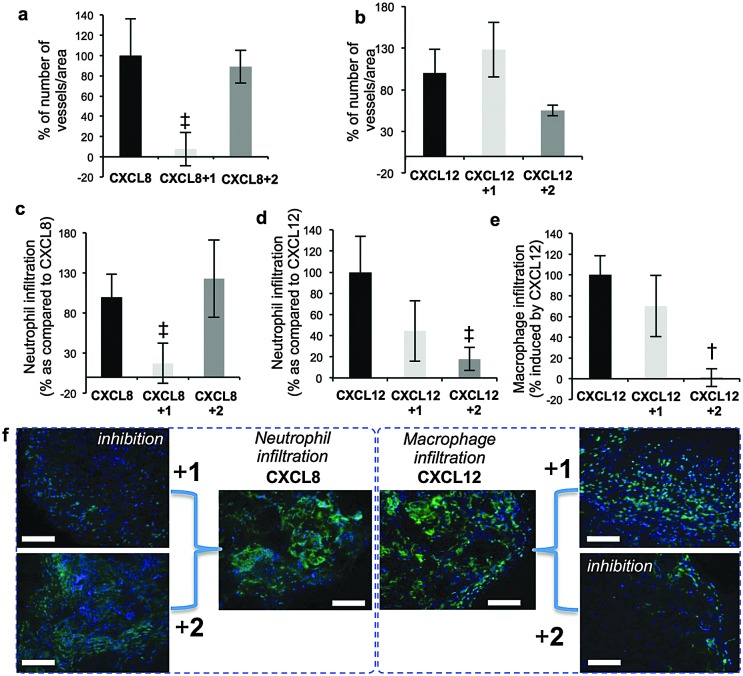
Differential effects of 12-mers 1 and 2 on CXCL8 and CXCL12 biological functions *in vivo*. (a and b) Effect on CXCL8/CXCL12-induced vascularization of sponges. (c and d) Effect on CXCL8/CXCL12 on neutrophil infiltration. (e) Effect on and CXCL12-induced accumulation of macrophages in sponges. [Number of blood vessels, neutrophils or macrophages per standardized sponge section as percentage for cytokine-impregnated sponges without oligosaccharides (100%)]. Data shown as mean ± SEM (*n* = 6). †, *p* < 0.01; ‡, *p* < 0.05. (f) Cell images for (c) and (e) – details ESI,† Fig. S24.

In summary, we demonstrate that an efficient iterative chemical strategy now extends to multigram scale synthesis of a site-specifically sulfated dodecasaccharide establishing this as a general gram-plus scale access to differentiated, structure-programmable synthetic dodecasaccharides. This has thereby also enabled the first *in vivo* studies with such pure longer H/HS oligosaccharides. Addition of a single 6-*O*-sulfate moiety to the non-reducing end of an otherwise homogeneously sulfated dodecasaccharide effects a switch between inhibition of CXCL12 and CXCL8, showing that sulfate location specificity can be a key determinant of biological effects. Such reversal of effects is not seen with a key control chemokine CCL19, and nor is it seen with angiogenic growth factors (VEGF, FGF2). These findings indicate that other significant site-specific sulfation-determined effects await discovery and biomedical exploitation.

Cancer Research UK (C2075/A9106) and the UK Medical Research Council (G0601746 and G902173) are thanked for project grant funding (GCJ and JMG). The EPSRC National Mass Spectrometry Service, Swansea are thanked for mass spectroscopic analyses, and Dr Matthew Cliff (MIB NMR Facility) and Rehana Sung (LCMS).

## References

[cit1] Kim S.-H., Turnbull J., Guimond S. (2011). J. Endocrinol..

[cit2] Zulueta M. M. L., Lin S.-Y., Hu Y.-P., Hung S.-C. (2013). Curr. Opin. Chem. Biol..

[cit3] Lindahl U., Kjellén L. (2013). J. Intern. Med..

[cit4] (b) Lico C.-H. Chang L. S. Huang T.-Y. Lin S.-Y. Chang C.-L. Arco S. D. Hung S.-C., Angew. Chem., Int. Ed., 2014, 53 , 9876 –9879 , and references therein . 10.1002/anie.201404154 25044485

[cit5] Hansen S. U., Baráth M., Salameh B. A., Pritchard R. G., Stimpson W. T., M Gardiner J., Jayson G. C. (2009). Orglett.

[cit6] Hansen S. U., Miller G. J., Baráth M., Broberg K. R., Avizienyte E., Jayson G. C., Gardiner J. M. (2012). J. Org. Chem..

[cit7] Hansen S. U., Miller G. J., Jayson G. C., Gardiner J. M. (2013). Orglett.

[cit8] Miller G. J., Hansen S. U., Cole C., Avizienyte E., Rushton G., Jayson G. C., Gardiner J. M. (2013). Chem. Sci..

[cit9] Nomenclature: Lawrence R. Lu H. Rosenberg R. D. Esko J. D. Zhang L., Nat. Methods, 2008, 5 , 291 –292 . 18376390 10.1038/nmeth0408-291

[cit10] Jastrebova N., Vanwildemeersch M., Lindahl U., Spillmann D. (2010). J. Biol. Chem..

[cit11] Naimy H., Buczek-Thomas J. A., Nugent M. A., Leymarie N., Zaia J. (2011). J. Biol. Chem..

[cit12] Xu Y., Cai C., Chandarajoti K., Hsieh P.-H., Li L., Pham T. Q., Sparkenbaugh E. M., Sheng J., Key N. S., Pawlinski R., Harris E. N., Linhardt R. J., Liu J. (2014). Nat. Chem. Biol..

[cit13] Handel T. M., Johnson Z., Crown S. E., Lau E. K., Proudfoot A. E. (2005). Annu. Rev. Biochem..

[cit14] Lira S. A., Furtado G. C. (2012). Immunol. Res..

[cit15] Zhang Q., Sun L., Yin L., Ming J., Zhang S., Luo W., Qiu X. (2013). Tumor Biol..

[cit16] Schlorke D., Thomas L., Samsonov S. A., Huster D., Arnhold J., Pichert A. (2012). Carbohydr. Res..

[cit17] Pichert A., Schlorke D., Franz S., Arnhold J. (2012). Biomatter.

[cit18] Ziarek J. J., Veldkamp C. T., Zhang F., Murray N. J., Kartz G. A., Liang X., Su J., Baker J. E., Linhardt R. J., Volkman B. F. (2013). J. Biol. Chem..

[cit19] Meyer B., Thunberg L., Lindahl U., Larm O., Leder I. G. (1981). Carbohydr. Res..

[cit20] Axelsson J., Xu D., Kang B. N., Nussbacher J. K., Handel T. M., Ley K., Sriramarao P., Esko J. D. (2012). Blood.

[cit21] Uchimura K., Morimoto-Tomita M., Bistrup A., Li J., Lyon M., Gallagher J., Werb Z., Rosen S. D. (2006). BMC Biochem..

[cit22] Jastrebova N., Vanwildemeersch M., Rapraeger A. C., Giménez-Gallego G., Lindahl U., Spillmann D. (2006). J. Biol. Chem..

[cit23] Ferreras C., Rushton G., Cole C., Babur M., Telfer B. A., van Kuppevelt T. H., Gardiner J. M., Williams K. J., Jayson G. C., Avizienyte E. (2012). J. Biol. Chem..

[cit24] de Paz J. L., Moseman E. A., Noti C., Polito L., von Andrian U. H., Seeberger P. H. (2007). Chem. Biol..

[cit25] Gama C. I., Tully S. E., Sotogaku N., Clark P. M., Rawat M., Vai-dehi N., Goddard W. A., Nishi A., Hsieh-Wilson L. C. (2006). Nat. Chem. Biol..

[cit26] Bishop J., Schuksz M., Esko J. D. (2007). Nature.

[cit27] Proudfoot A. E., Handel T. M., Johnson Z., Lau E. K., LiWang P., Clark-Lewis I., Borlat F., Wells T. N., Kosco-Vilbois M. H. (2003). Proc. Natl. Acad. Sci. U. S. A..

[cit28] Severin I. C., Soares A., Hantson J., Teixeira M., Sachs D., Valognes D., Scheer A., Schwarz M. K., Wells T. N. C., Proudfoot A. E. I., Shaw J. (2012). Front. Immunol..

