# Supplementary material for: Synthetic heparan sulfate dodecasaccharides reveal single sulfation site interconverts CXCL8 and CXCL12 chemokine biology
Source: Chem Commun (Camb). 2015 Aug 3;51(72):13846–9. doi: 10.1039/c5cc05222j (PMC4608306; doi:10.1039/c5cc05222j)
Supplement: Supplementary file 1 [file CC-051-C5CC05222J-s001.pdf]

Electronic Supplementary Material (ESI) for ChemComm.
This journal is © The Royal Society of Chemistry 2015

Supplementary Materials

Synthetic heparan sulfate dodecasaccharides reveal single sulfation site switch regulates CXCL8 and CXCL12 chemokine biology.

Authors: Gordon C. Jayson,[a] Steen U. Hansen,[b] Gavin J. Miller,[b] Claire L. Cole,[a] Graham Rushton,[a] Egle Avizienyte,[a] and John M. Gardiner[b*]

Affiliations:

[a] Institute of Cancer Sciences, University of Manchester, Cancer Research UK Manchester Institute, Wilmslow Road, Manchester M20 4BX, UK

[b] Manchester Institute of Biotechnology and the School of Chemistry, 131 Princess Street, The University of Manchester, Manchester M1 7DN, UK

* gardiner@manchester.ac.uk

Materials and Methods
1 Synthetic experimental and data
2 Biological Methods

Chemistry Data Figures (1-22)

Biology Figures (23-26)

Materials and Methods
1 Synthetic experimental and data

General methods

All the chemicals used were purchased from commercial sources without further purification. All reactions were monitored by TLC on Merck silica gel plates $^{60}F_{254}$. Silica gel 60 (particle size 0.035-0.070 mm) was used for column chromatography. ^{1}H NMR spectra were recorded at 800 or 400 MHz and ^{13}C spectra at 100 or 200 MHz respectively on Bruker DPX spectrometers. Mass spectra (MS) were recorded using a Micromass Platform II spectrometer using an electro spray ionization source or via the EPSRC National Mass Spectrometry Service (Swansea). Isotope patterns for compounds with mass > 1000 are included in the SI. Optical rotations were obtained using an AA-1000 polarimeter. Elemental analyses were performed by Micro Analytical Laboratory, School of Chemistry, The University of Manchester. NMR Data were reprocessed using iNMR 4 from Nucleomatica and Bruker Topspin.

Experimental procedures: Chemical synthesis

1.1 Methyl (2-azido-6-*O*-benzoyl-3-*O*-benzyl-2-deoxy-4-*O*-trichloroacetyl-α-D-glucopyranosyl)-(1→4)-(methyl 2-*O*-benzoyl-3-*O*-benzyl-α-L-idopyranosyl uronate)-(1→4)-2-azido-3,6-di-*O*-benzyl-2-deoxy-α-D-glucopyranosyl-(1→4)-(methyl 2-*O*-benzoyl-3-*O*-benzyl-α-L-idopyranosyl uronate)-(1→4)-2-azido-3,6-di-*O*-benzyl-2-deoxy- α-D-glucopyranosyl-(1→4)-(methyl 2-*O*-benzoyl-3-*O*-benzyl-α-L-idopyranosyl uronate)-(1→4)-2-azido-3,6-di-*O*-benzyl-2-deoxy-α-D-glucopyranosyl-(1→4)-(methyl 2-*O*-benzoyl-3-*O*-benzyl-α-L-idopyranosyl uronate)-(1→4)-2-azido-3,6-di-*O*-benzyl-2-deoxy-α-D-glucopyranosyl-(1→4)-(methyl 2-*O*-benzoyl-3-*O*-benzyl-α-L-idopyranosyl uronate)-(1→4)-2-azido-3,6-di-*O*-benzyl-2-deoxy-α-D-glucopyranosyl-(1→4)-(methyl 2-*O*-benzoyl-3-*O*-benzyl-α-L-idopyranoside) uronate (Supplementary compound S1)

Acceptor decasaccharide **4** (5.07 g, 1.34 mmol) and thioglycoside disaccharide donor **5** (1.36 g, 1.34 mmol) was dissolved in dry DCM (25 mL) under N_2. Freshly activated 4Å powdered molecular sieves (1.0 g) was added and the solution cooled to 0 °C in an icebath. After 10 min. NIS (0.60 g, 2.67 mmol) was added, and after another 10 min. AgOTf (catalytic amount) was added. The suspension changed colour from pale yellow to deep red, was stirred for 45 min. and the reaction was quenched into a separating funnel containing a mixture of DCM (200 mL), saturated aqueous $NaHCO_3$ (200 mL) and $Na_2S_2O_3$ (10 mL, 10% aqueous). After shaking until the iodine colour was removed the suspension was filtered through a short pad of Celite® washing with water and DCM. The layers were separated and the aqueous extracted with DCM (50 mL). The organic layers were combined, dried ($MgSO_4$) and solvent removed *in vacuo*. The crude was purified by silica gel flash column chromatography (toluene/acetone gradient 30:1 to 10:1) to give **S1** (5.25 g, 84%) as a white foam. *Rf* 0.20 (EtOAc/hexane 3:5). $[\alpha]_D^{20}$ = +36.4 (*c* = 0.14, CH_2Cl_2). ^{1}H NMR (400 MHz; $CDCl_3$) δ 8.16-8.07 (m, 6H, Bz), 8.02-7.96 (m, 8H, Bz), 7.59-7.06 (m, 106H, Ph), 5.63-5.59 (m, 5H, H_{CEGIK}-1), 5.35 (t, *J* = 9.6 Hz, 1H, H_L-4), 5.27-5.20 (m, 5H, H_{CEGIK}-2), 5.14-5.13 (m, 1H, H_A-2), 5.10-5.09 (m, 1H, H_A-1), 5.02-4.93 (m, 5H, H_{BDFHJ}-1), 4.88-4.37 (m, 41H, H_L-1, H_{ACEGIK}-5, 16xCH_2Ph), 4.30-4.21 (m, 7H, H_{CEGIK}-3, CH_2Ph), 4.14-3.32 (m, 42H, H_A-3, H_{ACEGIK}-4, $H_{BDFHJ}{}^{L}$-2, H_{BDFHJL}-3, H_{BDFHJ}-4, H_{BDFHJL}-5, H_{BDFHJL}-6$_{ab}$), 3.54 (s, 3H, COOCH_3), 3.54 (s, 3H, COOCH_3), 3.51 (s, 3H, COOCH_3), 3.39 (s, 3H, COOCH_3), 3.35 (s, 3H, COOCH_3), 3.33 (s, 3H, COOCH_3), 3.29 (s, 3H, OCH_3). ^{13}C NMR (100 MHz; $CDCl_3$): δ 169.6, 169.3, 169.3, 169.3, 165.9, 165.6, 165.3, 165.2,

165.2, 138.1, 138.0, 137.9, 137.9, 137.8, 137.6, 137.5, 137.4, 137.4, 137.3, 136.6, 133.7, 133.5, 133.3, 130.0, 130.0, 129.9, 129.9, 129.8, 129.6, 129.5, 129.4, 129.4, 129.3, 129.3, 129.1, 128.9, 128.8, 128.7, 128.6, 128.5, 128.5, 128.4, 128.3, 128.2, 128.2, 128.1, 128.1, 128.0, 127.8, 127.7, 127.7, 127.7, 127.5, 127.5, 127.3, 127.2, 125.4, 100.3, 99.6, 99.3, 99.2, 98.5, 98.2, 98.1, 98.0, 98.0, 89.5, 78.3, 78.2, 78.1, 78.1, 77.6, 77.4, 76.0, 75.9, 75.9, 75.9, 75.8, 75.8, 75.6, 75.5, 75.5, 75.4, 75.4, 75.4, 75.4, 75.1, 74.6, 74.4, 74.4, 74.3, 74.2, 74.1, 74.0, 74.0, 73.7, 73.7, 73.5, 72.5, 71.5, 71.4, 71.3, 71.2, 70.7, 70.7, 70.7, 70.6, 70.5, 69.8, 68.9, 68.3, 68.0, 67.8, 67.4, 67.3, 67.3, 63.4, 63.3, 63.1, 63.0, 61.3, 56.3, 53.5, 52.2, 52.1, 51.8, 51.7, 51.7, 51.7. MALDI TOF: m/z: calcd for $C_{249}H_{247}Cl_3N_{18}NaO_{69}$ $[M+Na]^+$: 4720.5; found: 4720.6. Elemental analysis calcd (%) for $C_{249}H_{247}Cl_3N_{18}O_{69}$: C 63.60, H 5.29, N 5.36; found C 63.68, H 5.32, N 5.32.

1.2 Methyl 2-azido-3-*O*-benzyl-2-deoxy-α-D-glucopyranosyl-(1→4)-3-*O*-benzyl-α-L-idopyranosyl uronic acid-(1→4)-2-azido-3,6-di-*O*-benzyl-2-deoxy-α-D-glucopyranosyl-(1→4)-3-*O*-benzyl-α-L-idopyranosyl uronic acid-(1→4)-2-azido-3,6-di-*O*-benzyl-2-deoxy-α-D-glucopyranosyl-(1→4)-3-*O*-benzyl-α-L-idopyranosyl uronic acid-(1→4)-2-azido-3,6-di-*O*-benzyl-2-deoxy-α-D-glucopyranosyl-(1→4)-3-*O*-benzyl-α-L-idopyranosyl uronic acid-(1→4)-2-azido-3,6-di-*O*-benzyl-2-deoxy-α-D-glucopyranosyl-(1→4)-3-*O*-benzyl-α-L-idopyranosyl uronic acid-(1→4)-2-azido-3,6-di-*O*-benzyl-2-deoxy-α-D-glucopyranosyl-(1→4)-3-*O*-benzyl-α-L-idopyranoside uronic acid (6)

The dodecasaccharide **S1** (5.19 g, 1.10 mmol) was dissolved in THF (125 mL) and MeOH (50 mL) and then cooled to 0 °C in an icebath. Then $LiOH.H_2O$ (1.20 g, 28.7 mmol) was dissolved in 50 mL water and added in portions over 25 min. (5x5 mL) and the rest after 12 h. The solution was stirred for a total of 4 h at 0 °C and then at room temperature for another 10 h. The solution was then extracted with EtOAc (300 mL, 100 mL) and HCl (0.2 M, 250 mL). The combined organic phase was washed with brine (100 mL), dried ($MgSO_4$), filtered and evaporated. The crude was purified using flash column chromatography (DCM/MeOH gradient 50:1 to 30:1 to 20:1). This yielded the carboxylic acid dodecasaccharide **6** (3.27 g, 79%) as a white solid. R_f 0.31 (DCM/MeOH 10:1). ^1H NMR (400 MHz; CD_3OD) δ 7.41-7.12 (m, 85H, Ph), 5.40-3.30 (m, 106H, $H_{ABCDEFGHIJKL}$-1, $H_{ABCDEFGHIJKL}$-2, $H_{ABCDEFGHIJKL}$-3, $H_{ABCDEFGHIJKL}$-4, $H_{ABCDEFGHIJKL}$-5, H_{BDFHJL}-6_{ab} , 17xCH_2Ph), 3.42 (s, 3H, OCH_3). FT MS: m/z: calcd for $C_{192}H_{206}N_{18}O_{61}$ $[M-2H]^{2-}$: 1869.6791; found: 1869.6783.

1.3 Methyl 2-azido-3-*O*-benzyl-2-deoxy-4,6-di-*O*-sulfonato-α-D-glucopyranosyl-(1→4)-3-*O*-benzyl-2-*O*-sulfonato-α-L-idopyranosyl uronic acid-(1→4)-2-azido-3,6-di-*O*-benzyl-2-deoxy-α-D-glucopyranosyl-(1→4)-3-*O*-benzyl-2-*O*-sulfonato-α-L-idopyranosyl uronic acid-(1→4)-2-azido-3,6-di-*O*-benzyl-2-deoxy-α-D-glucopyranosyl-(1→4)-3-*O*-benzyl-2-*O*-sulfonato-α-L-idopyranosyl uronic acid-(1→4)-2-azido-3,6-di-*O*-benzyl-2-deoxy-α-D-glucopyranosyl-(1→4)-3-*O*-benzyl-2-*O*-sulfonato-α-L-idopyranosyl uronic acid-(1→4)-2-azido-3,6-di-*O*-benzyl-2-deoxy-α-D-glucopyranosyl-(1→4)-3-*O*-benzyl-2-*O*-sulfonato- α-L-idopyranosyl uronic acid-(1→4)-2-azido-3,6-di-*O*-benzyl-2-deoxy-α-D-glucopyranosyl-(1→4)-3-*O*-benzyl-2-*O*-sulfonato-α-L-idopyranoside uronic acid octa sodium salt (7)

The dodecasaccharide **6** (3.24 g, 0.87 mmol) was dissolved in dry pyridine (150 mL), pyridine sulfurtrioxide complex (3.30 g, 20.8 mmol) added and then heated to 50 °C for 14 h while kept under N_2. The reaction was quenched with aqueous $NaHCO_3$ (4.3 g, 51.9 mmol, in 50 mL H_2O) and evaporated. The crude was redissolved in MeOH/DCM (50 mL/50 mL), filtered and evaporated. This residue was then purified using flash column chromatography (DCM/MeOH + 1% HCOOH

gradient 8:1 to 7:1 to 5:1 or DCM/MeOH + 1% NH_4OH (35% aq.) gradient 5:1 to 4:1 to 3:1). This yielded the (mono 4S,6S), 2S dodecasaccharide **S5** (3.35 g, 85%) as a glassy solid. R_f 0.16 (DCM/MeOH 3:1 + 1% HCOOH). $[\alpha]_D^{20}$ = +24.7 (*c* = 1.35, MeOH). ^{1}H NMR (400 MHz; CD_3OD) δ 7.48-7.09 (m, 85H, Ph), 5.87-3.40 (m, 106H, $H_{ABCDEFGHIJKL}$-1, $H_{ABCDEFGHIJKL}$-2, $H_{ABCDEFGHIJKL}$-3, $H_{ABCDEFGHIJKL}$-4, $H_{ABCDEFGHIJKL}$-5, H_{BDFHJL}-6$_{ab}$, 17xCH_2Ph), 3.45 (s, 3H, OCH_3). FT MS: m/z: calcd for $C_{196}H_{213}N_{19}O_{85}S_8$ [*M*-8Na+2H+Et_2NH]$^{6-}$: 741.6799; found: 741.6771 (The MS sample was ion exchanged with Amberlite® IRC 86 NH_4^+ resin).

1.4 Methyl 4,6-di-*O*-sulfonato-α-D-glucosaminopyranosyl-(1→4)-2-*O*-sulfonato-α-L-idopyranosyl uronic acid-(1→4)-α-D-glucosaminopyranosyl-(1→4)-2-*O*-sulfonato-α-L-idopyranosyl uronic acid-(1→4)-α-D-glucosaminopyranosyl-(1→4)-2-*O*-sulfonato-α-L-idopyranosyl uronic acid-(1→4)-α-D-glucosaminopyranosyl-(1→4)-2-*O*-sulfonato-α-L-idopyranosyl uronic acid-(1→4)-α-D-glucosaminopyranosyl-(1→4)-2-*O*-sulfonato-α-L-idopyranosyl uronic acid-(1→4)-α-D-glucosaminopyranosyl-(1→4)-2-*O*-sulfonato-α-L-idopyranoside uronic acid octa sodium salt 8

The dodecasaccharide **7** (2.96 g, 0.65 mmol) was dissolved in a mixture of MeOH/H_2O (170 mL/85 mL) and $NaHCO_3$ (327 mg, 3.89 mmol). Then a three way tap was attached to the flask and fitted with a nitrogen balloon and the other tap was attached to a water aspirator vacuum. Switching between vacuum and nitrogen balloon 5 times ensured removal of all oxygen from flask and solvent. Then was added $Pd(OH)_2$/C (1.12 g, 10-20%) and again flushed with nitrogen. The nitrogen balloon was replaced with a hydrogen balloon and the flask again subjected to vacuum and hydrogen 5 times to ensure all the nitrogen was replaced with hydrogen. The reaction was stirred vigorously for 48, then more $Pd(OH)_2$/C catalyst added (0.55 g) and stirred another 36 h. The product mixture was partially evaporated to leave an aqueous solution that was filtered through Celite®→ and washed with water (3x10 mL). The combined filtrate was then evaporated and purified by a short Sephadex→ G-25 column to give dodecasaccharide amine **8** (1.90 g, 97%) as a glassy solid.

$[\alpha]_D^{20}$ = +62.5 (*c* = 2.10, H_2O). ^{1}H NMR (400 MHz; D_2O) δ 5.41-5.36 (m, 6H, H_{BDFHJL}-1), 5.29-5.20 (m, 5H, H_{CEGIK}-1), 5.01-5.00 (m, 1H, H_A-1), 4.91-4.88 (m, 5H, H_{CEGIK}-5), 4.51-4.50 (m, 1H, H_A-5), 4.36-4.20 (m, 15H, H_{ACEGIK}-2, H_{ACEGIK}-3, H_L-4, H_L-6$_{ab}$), 4.16-4.09 (m, 6H, H_{ACEGIK}-4), 4.05-3.88 (m, 6H, H_{BDFHJL}-3, H_L-5), 3.89-3.78 (m, 15H, H_{BDFHJ}-5, H_{BDFHJ}-6$_{ab}$), 3.77-3.70 (m, 5H, H_{BDFHJ}-4), 3.44-3.39 (m, 1H, H_L-2), 3.39 (s, 3H, OMe), 3.35-3.30 (m, 5H, H_{BDFHJ}-2). FT MS: m/z: calcd for $C_{73}H_{112}N_6O_{85}S_8$ [*M*-8Na+2H]$^{6-}$: 448.0404; found: 448.0410 (The MS sample was ion exchanged with Amberlite® IRC 86 NH_4^+ resin).

1.5 Methyl 4,6-di-*O*-sulfonato-2-*N*-sulfonato-α-D-glucosaminopyranosyl-(1→4)-2-*O*-sulfonato-α-L-idopyranosyl uronate-(1→4)-2-*N*-sulfonato-α-D-glucosaminopyranosyl-(1→4)-2-*O*-sulfonato-α-L-idopyranosyl uronate-(1→4)-2-*N*-sulfonato-α-D-glucosaminopyranosyl-(1→4)-2-*O*-sulfonato-α-L-idopyranosyl uronate-(1→4)-2-*N*-sulfonato-α-D-glucosaminopyranosyl-(1→4)-2-*O*-sulfonato-α-L-idopyranosyl uronate-(1→4)-2-*N*-sulfonato-α-D-glucosaminopyranosyl-(1→4)-2-*O*-sulfonato-α-L-idopyranosyl uronate-(1→4)-2-*N*-sulfonato-α-D-glucosaminopyranosyl-(1→4)-2-*O*-sulfonato-α-L-idopyranoside uronate icosa sodium salt (2)

The dodecasaccharide **4** (1.90 g, 0.63 mmol) was dissolved in water (70 mL), $NaHCO_3$ (1.91 g, 22.7 mmol) and pyridine sulfur trioxide complex (1.81 g, 11.4 mmol) was added with vigorous stirring. This procedure was repeated after 1 h, 2.5 h, 4 h, 6 h and 18 h ($NaHCO_3$: 1.95 g, 1.96 g, 1.94 g, 1.92 g, 2.03 g. Py.SO_3: 1.79 g, 1.77 g, 1.82 g, 1.70 g, 1.75 g). After 20 h the mixture was

evaporated. The crude containing Na_2SO_4 salts was redissolved in minimum amount of water and purified by passage through a Sephadex G-25 column (40 mL) by eluting with water (the procedure was repeated three times). The combined fractions containing oligosaccharide were pooled and evaporated to yield **2** (1.97 g, 87%) as a glassy solid. $[\alpha]_D^{20}$= +59.6 (c = 0.95, H_2O). ^{1}H NMR (400 MHz; D_2O) δ 5.35-5.18 (m, 11H, $H_{BCDEFGHIJKL}$-1), 5.03-5.02 (m, 1H, H_A-1), 4.93-4.87 (m, 5H, H_{CEGIK}-5), 4.44 (d, 1H, J = 2.0 Hz, H_A-5), 4.33-4.18 (m, 15H, H_{ACEGIK}-2, H_{ACEGIK}-3, H_L-4, H_L-6$_{ab}$), 4.06-3.97 (m, 8H, H_{ACEGIK}-4, H_L-3, H_L-5), 3.89-3.75 (m, 15H, H_{BDFHJ}-5, H_{BDFHJ}-6$_{ab}$), 3.74-3.61 (m, 10H, H_{BDFHJ}-3, H_{BDFHJ}-4), 3.39 (s, 3H, OMe), 3.31 (dd, 1H, J = 10.8 Hz, 3.2 Hz, H_L-2), 3.24-3.16 (m, 5H, H_{BDFHJ}-2). ^{13}C NMR (201 MHz; H_2O+D_2O) δ 175.3, 175.0, 100.1, 99.6, 99.2, 97.4, 97.2, 96.9, 77.4, 76.8, 75.7, 74.9, 74.5, 74.3, 72.4, 71.5, 71.0, 69.5, 68.9, 68.7, 68.3, 68.1, 67.8, 67.5, 67.4, 66.9, 66.2, 59.9, 59.7, 59.3, 58.4, 58.2, 58.0, 57.5, 57.3, 55.5, 55.4, 54.4. FT MS: m/z: calcd for $C_{73}H_{111}N_6O_{103}S_{14}$ $[M+13H-20Na]^{7-}$: 452.4251; found: 452.4239 (The MS sample was ion exchanged with Amberlite® IRC 86 NH_4^+ resin).

2 Biology Methods
Experimental procedures: Biology

2.1 Cell culture.

HUVECs (Lonza) were cultured in EBM-2 medium supplemented with SingleQuot growth supplements (Lonza) up to passage 7. Generation of MCF-10A cells (ATCC) expressing CXCR4 (MCF-10A-CXCR4) has been described[1]. Cells were cultured in DMEM/F12 (Invitrogen) supplemented with 5% horse serum (Invitrogen), 20 ng/mL EGF (R&D Systems), 0.5 µg/mL hydrocortisone (Sigma), 100 ng/mL cholera toxin (Sigma), insulin 10 µg/mL (Sigma) and 1x penicillin/streptomycin solution (Invitrogen).

2.2 HS competition assay.

HS competition assays were performed as previously described[2]. Briefly, ninety six-well heparin-binding plates (Iduron) were coated with HS (Iduron). After blocking and washes, 10 ng of IL-8 or SDF-1α (both from R&D Systems) were diluted in 100 µL of standard assay buffer (50 mM sodium acetate pH 7.3, 150 mM sodium chloride and 0.2% Tween20) containing 10 mg/mL BSA. Oligosaccharides were mixed at 0.1-100 µg/mL range for both IL-8 and SDF-1α and added to immobilized HS for 2 hours. After washes, HRP-tagged primary antibodies against IL-8 (R&D Systems, Quantikine ELISA kit) or SDF-1α (R&D Systems, Quantikine ELISA kit) were added to the wells for 2 hours. Following the washes, TMB solution (Sigma-Aldrich) was added for 30 min and the reaction was stopped with 2M sulphuric acid. Optical density was measured at 450 nm.

2.3 *In vitro* assays for testing angiogenic functions of endothelial cells.

HUVEC migration in the presence or absence of oligosaccharides was tested using a wound healing assay[2]. Briefly, $2X10^4$ HUVECs were seeded per well of a 96-well plate and incubated at +37 ˚C, in 5% CO_2. Confluent monolayers were serum-starved in EBM-2 medium lacking supplements containing 0.1% FBS for 24 hours. Cell monolayers were wounded with a pipette tip and overlayed with EBM-2 medium without supplements with 0.1% FBS or medium supplemented with IL-8 (50 ng/mL) or SDF1-α (50 ng/mL; both from R&D Systems). Synthetic oligosaccharides were administered at 1, 10 and 50 µg/ml. Phase contrast images were taken immediately after beginning the treatment and after 24 hours using Zeiss Axiovert 200M microscope (Zeiss, Hertfordshire, UK) enclosed in a full environmental chamber. The unpopulated areas at the beginning and the end of

each experiment were measured using MetaMorph image analysis software (Molecular Devices). The cell advancement area was derived for each treatment.

2.4 Chemotaxis assay.

The transwell migration assay was performed using 24-well transwell units with 8 μm pore size PVDF polycarbonate uncoated filters (Costar). MCF10A-CXCR4 cells ($5x10^4$) were seeded in serum-free DMEM/F12 in the upper compartment of the chemotaxis chambers. DMEM/F12 supplemented with 5% horse serum containing either recombinant SDF-1α (100 ng/mL; R&D Systems) or PBS as control was added to the lower well to stimulate directional migration of MCF10A-CXCR4 cells. Oligosaccharides were used at 50 μg/mL concentration. MCF10A-CXCR4 cells were allowed to migrate for 24 h at 37 ºC in a tissue culture incubator. The cells remaining on the upper surface of the membrane were removed, and those present on the lower surface of the filter were fixed in 4% PFA for 5 min, stained with DAPI and counted at x10 magnification using Olympus BX51 microscope. Twenty independent fields per filter were analysed.

2.5 Gelfoam *in vivo* angiogenesis assay.

All procedures were carried out in accordance with UKCCCR guidelines 1999 by approved protocol (Home Office Project license no. 40-3609). Sterile absorbable sponges (Pharmacia, Peapack, NJ) were cut into 5 × 5 × 7-mm pieces and hydrated overnight at 4 °C in sterile PBS. Excess PBS was then adsorbed onto sterile filter paper. The sponges were then soaked with 0.4% agarose (100 μl) containing either saline, 250 ng of CXCL8 (R&D Systems), 250 ng of CXCL12 (PreproTech) or chemokines plus each oligosaccharide (250 μg). The agarose-Gelfoam plugs were then allowed to harden for 1 hour at room temperature before s.c. implantation into mice. Female SCID-bg mice (Paterson Institute, The University of Manchester) were anesthetized with isoflurane, and the plugs were implanted s.c. via a midline incision of the abdominal skin and placed either toward the right flank or the left flank. Each group consisted of 6 mice. After seven days gelfoam plugs were harvested, placed in OCT solution, and snap-frozen in liquid nitrogen for subsequent immunohistochemical analyses. The sections were stained with anti-mouse CD31 as described[3]. Monoclonal anti-α-SMA (clone 1A4) Cy3 conjugate (Sigma-Aldrich) diluted 1:50 was added to visualize mural cells. Neutrophils were visualized with anti-Ly-6B.2 alloantigen antibody (clone 7/4; AbD Serotec) diluted 1:100 and incubated overnight at 4 ºC. AlexaFluor488–conjugated donkey anti-rat IgG antibody (1:1000; Invitrogen) was used as a secondary antibody. Macrophage specific anti-receptor F4/80 rat monoclonal antibody (AbD Serotec) was used at 1:1000 dilution and secondary AlexaFluor488-conjugated donkey anti-rat antibody (Invitrogen) was used at 1:1000. Images were collected with 3D-Histech Mirax scanner (3DHISTECH), viewed with Pannoramic Viewer software (3DHISTECH) and analysed by ImageJ software for CD31-positive blood vessels, neutrophil and macrophage staining. The whole area in each sponge section was evaluated using Metamorph image analysis software (Molecular Devices). The number of blood vessels in each analysed section was normalized to the standardized section area.

2.6 Leukocyte Transmigration Assay

Leukocyte transmigration through HUVEC monolayer in response to chemokines CXCL12 CXCL8 and CCL19 (MIP-3β) was tested using the CytoSelect Leukocyte Transmigration Assay (Cell Biolabs, Cambridge, UK) following the manufacturer's protocol.

Briefly, $7x10^5$ HUVEC in EBM-2 medium containing all supplements were added to each insert, which was placed in a 24-well plate containing 500 μl of complete EBM-2 medium. HUVEC were cultured for 30 hours following addition of 40 ng/ml of TNFα for a further 18 hours.

Twenty ml of peripheral blood was collected from healthy volunteers and diluted with 20 ml of HBSS lacking calcium and magnesium (Fisher Scientific, Loughborough, UK). Fifteen ml of Ficoll-Paque Plus solution (GE Healthcare, Uppsala, Sweden) was added. After centrifugation at 400 g for 35 minutes at room temperature, buffy coats were collected, aliquoted and frozen for long-term storage in liquid nitrogen.

When required, buffy coats were cultured for 24 hours in RPMI medium containing glutamine and supplemented with 10% FBS. Cells were collected by centrifugation at 400 g for 5 minutes, resuspended at $1x10^6$/ml and LeukoTracker staining solution was added at 1:500 for 1 hour. Cells were washed 3 times with RPMI containing 0.5% FBS and resuspended in RPMI containing 10% FBS. The inserts were removed from EBM-2 medium and placed in RPMI 10% FBS that contained CXCL12, CXCL8 or CCL19 at 1 μg/ml concentration or a mix of each chemokine and respective oligosaccharide at 1 mg/ml concentration. Oligosaccharide and chemokines were pre-incubated for 2 hours before adding to the inserts containing a HUVEC monolayer. Leukocytes ($3x10^5$) were added to the upper chamber and maintained there for 2 hours in tissue culture incubator. Transmigrated leukocytes were lysed in solution and fluorescence was measured at 480/520nm.

2.7 Immunoblotting.

HUVECs were plated in 6-well plates at 1 x 10^5 cells/well in EBM-2 medium lacking SingleQuot growth supplements and containing 0.1% FBS. After 18 hours starvation, cells were stimulated with IL-8 (50 ng/ml) for 10 minutes or SDF-1α (50 ng/ml) for 10 minutes in the presence or absence of oligosaccharides. Cell lysate preparation and immunoblotting was performed as described[2]. Mouse monoclonal anti-phospho-ERK1/2 (Thr202/Tyr204; clone E10) antibody was used at 1:2000, rabbit polyclonal anti-ERK1/2 at 1:500, mouse monoclonal anti-phospho-STAT3 (Ser727; clone 6E4; all from Cell Signaling Technology) at 1:500, and anti-GAPDH antibody (Abcam) at 1:2000. After washes, the membranes were incubated with horseradish peroxidase-conjugated goat anti-rabbit or anti-mouse IgG (Sigma-Aldrich) diluted 1:5000 in 5% nonfat dried milk in PBST and bands were visualized by chemiluminescence (PerkinElmer, MA, USA). Intensity of bands was evaluated by densitometry analysis, followed by normalization against loading controls.

2.8 Statistical analysis.

Data are expressed as the mean ±SEM. For comparison of groups, the two-tailed Student's t test was used. A level of $P < 0.05$ was considered as statistically significant.

References

1. Elenbaas, B. *et al. Genes Dev*. **15**, 50-65, (2001)**.**
2. Cole, C.L. *et al. PLOS ONE* **5**, e11644 (2010).
3. Ferreras, C., et al. *J. Biol. Chem.* **287**, 36132-36146, (2012).

Chemistry Data Figures

Supplementary Figure 2: ^{1}H NMR (400 MHz; $CDCl_3$) spectrum for **S1**

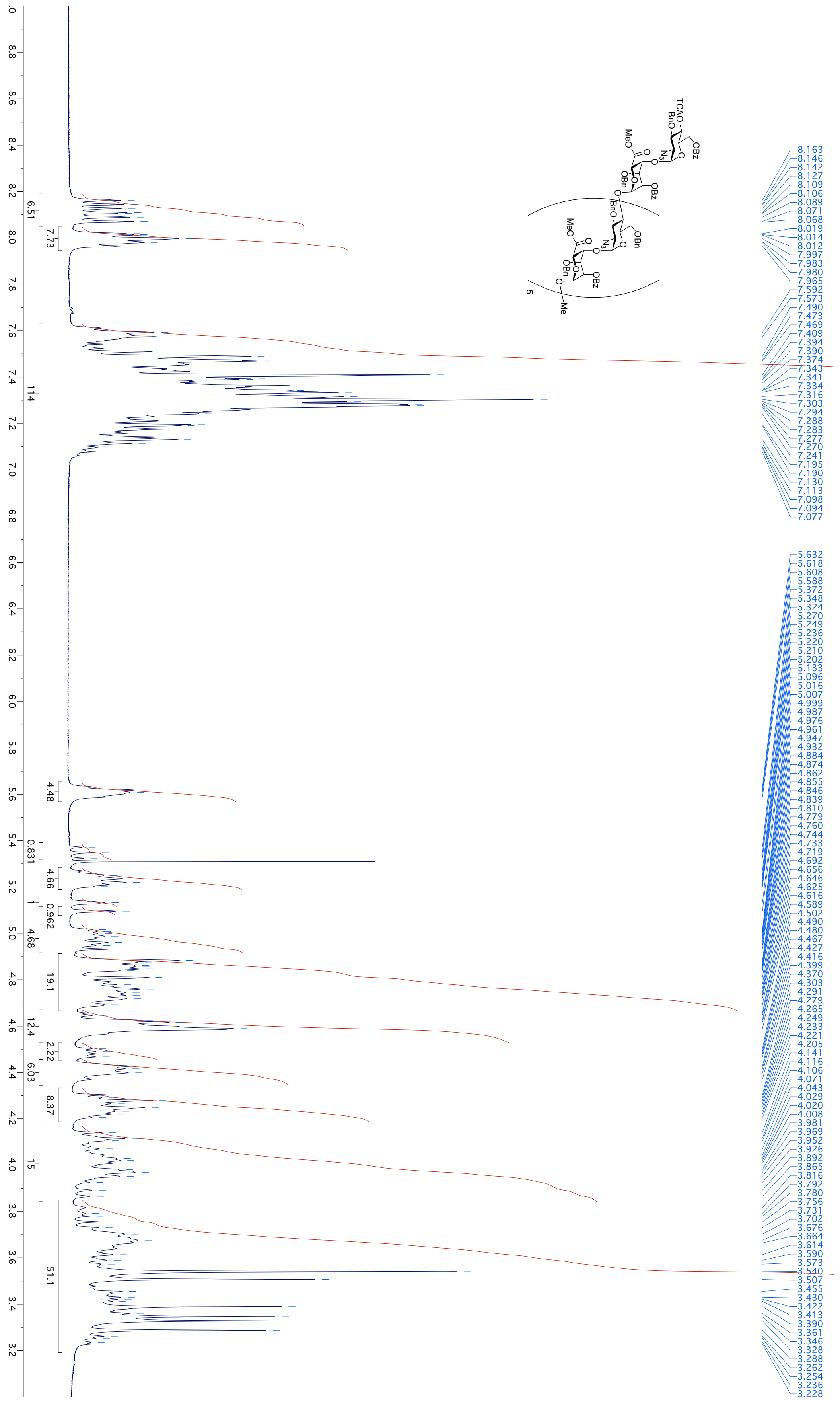

Supplementary Figure 3: ^{1}H NMR (400 MHz; $CDCl_3$) spectrum expansion for **S1**

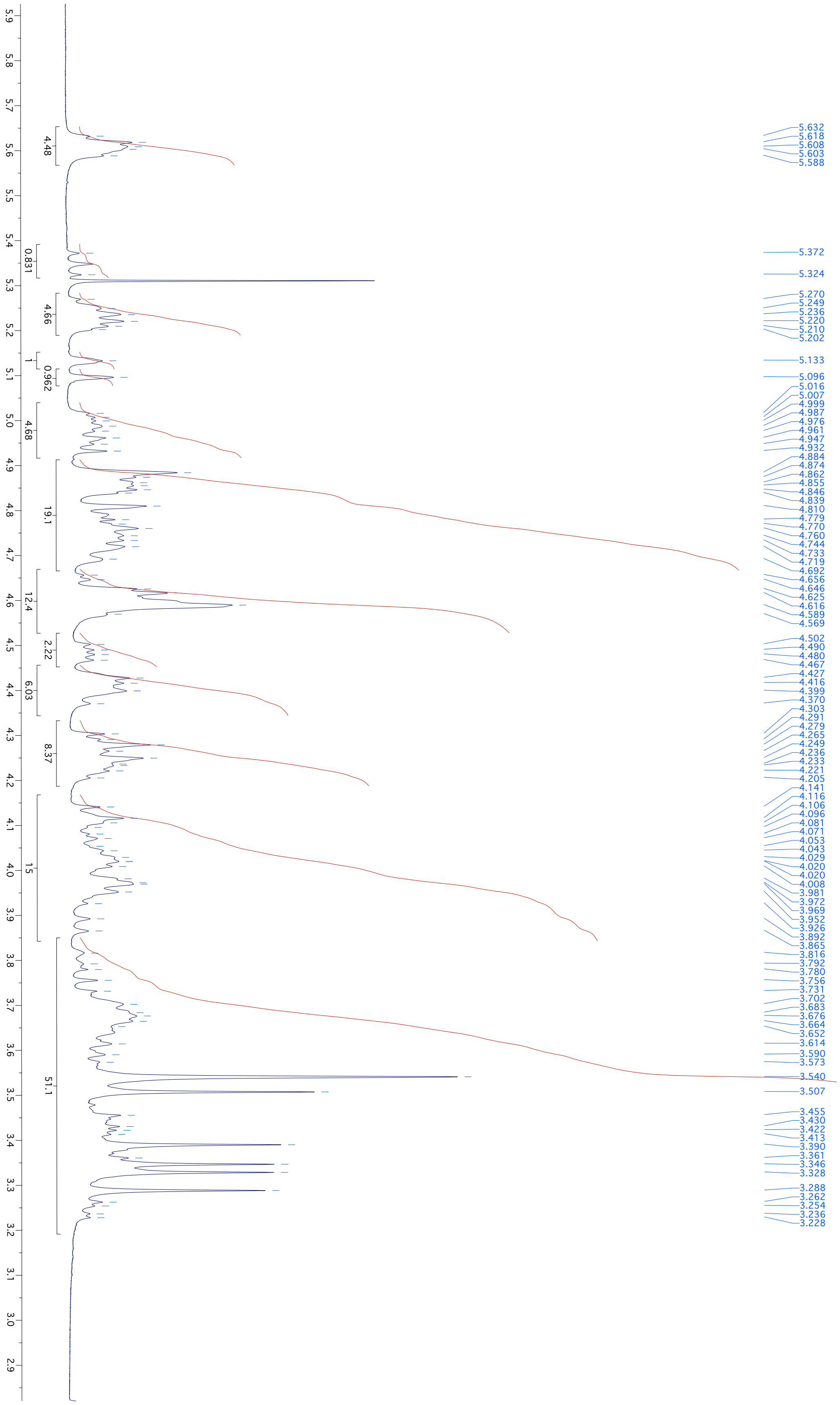

Supplementary Figure 4: HMQC (400 MHz; $CDCl_3$) NMR spectrum for **S1**

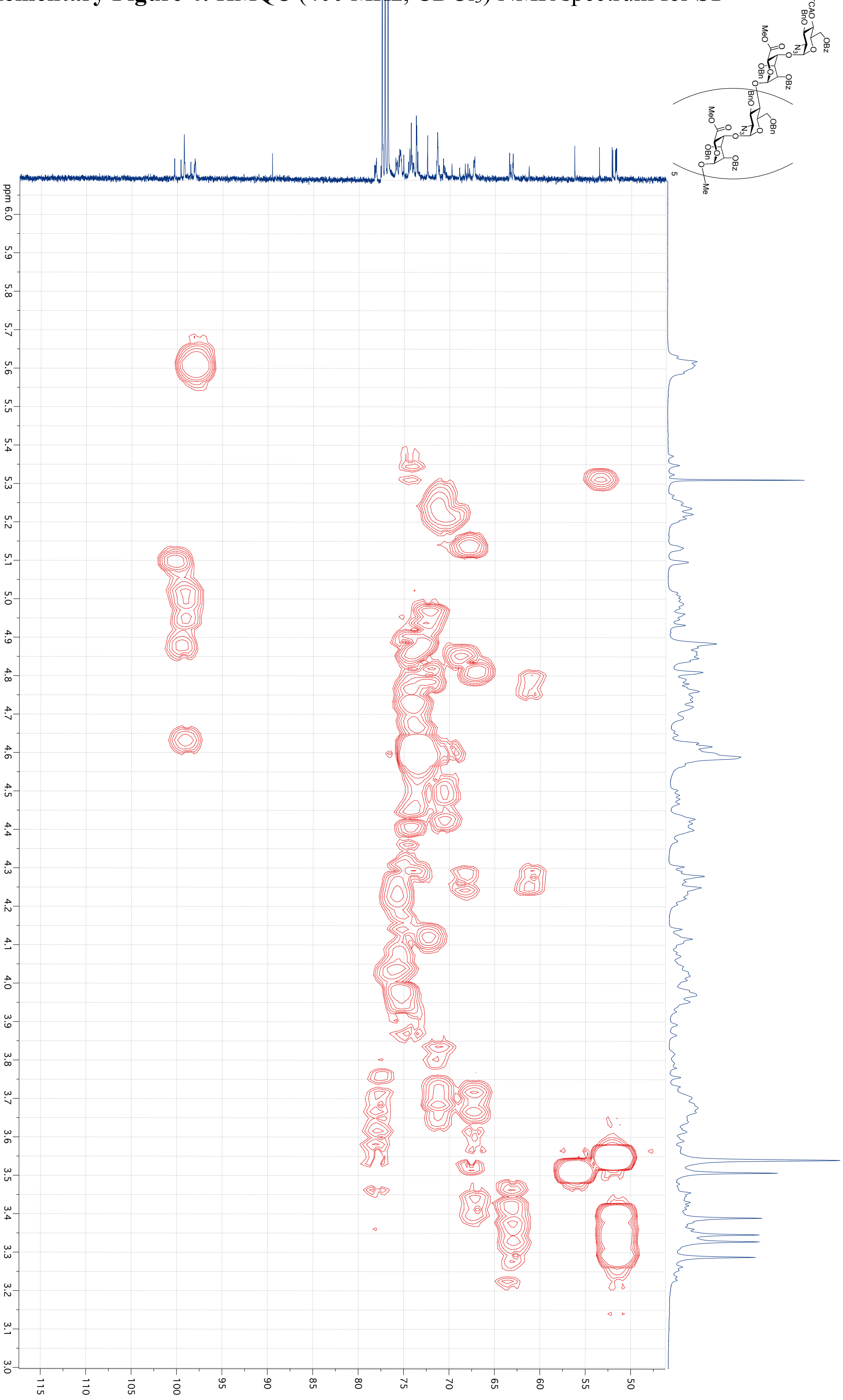

Supplementary Figure 5: ^{13}C NMR (100 MHz; $CDCl_3$) spectrum for **S1**

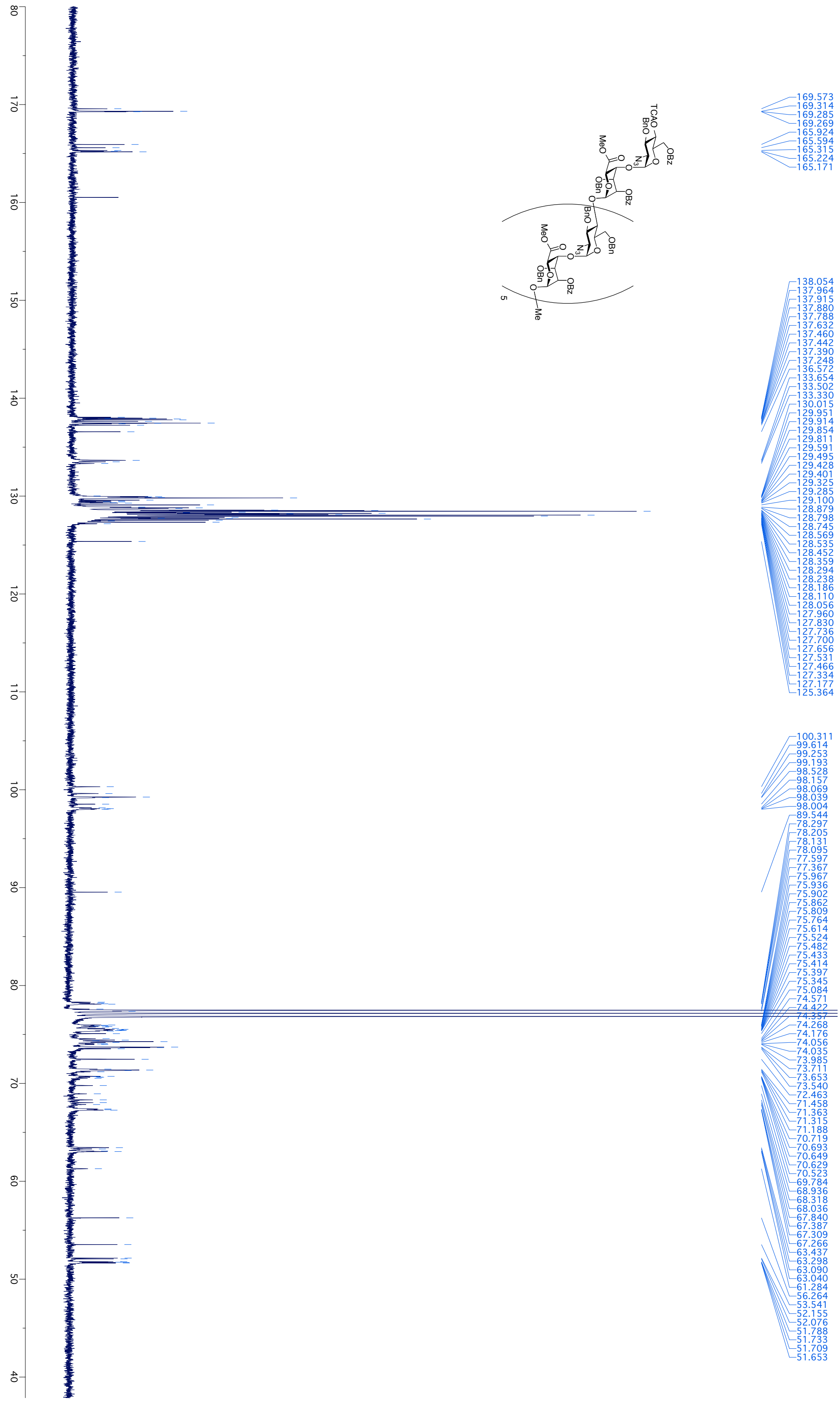

Supplementary Figure 6: LCMS for **S1**

Display Report

Analysis Info

Analysis Name	SU 1491 3CM COL 16TH JULY.D	Acquisition Date	16/07/2013 13:04:44
Method	GAVIN MILLER 3CM COL 100ACN.M	Operator	mib
Sample Name	SU 1491 3cm col 16th July	Instrument	LC-MSD-Trap-SL
Comment	Gavin Miller SU 1491 16th July<br>Supelco C8 4.6x30mm 5u 100% ACN 5ul inj<br>ESI source +ve ES 230nm 0.5ml/min		

Acquisition Parameter

Ion Source Type	ESI	Ion Polarity	Positive	Alternating Ion Polarity	off
Mass Range Mode	Extended	Scan Begin	1000 m/z	Scan End	4000 m/z
Capillary Exit	280.0 Volt	Skim 1	40.0 Volt	Trap Drive	218.9
Accumulation Time	96865 µs	Averages	7 Spectra	Auto MS/MS	off

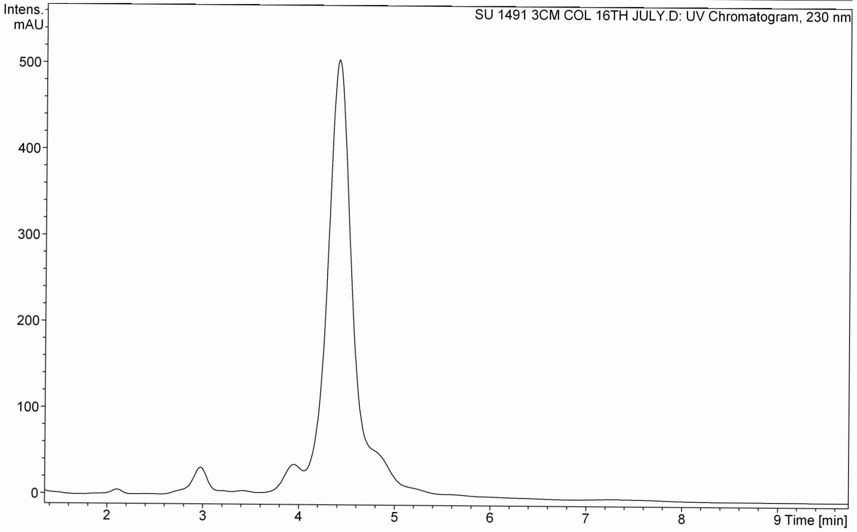

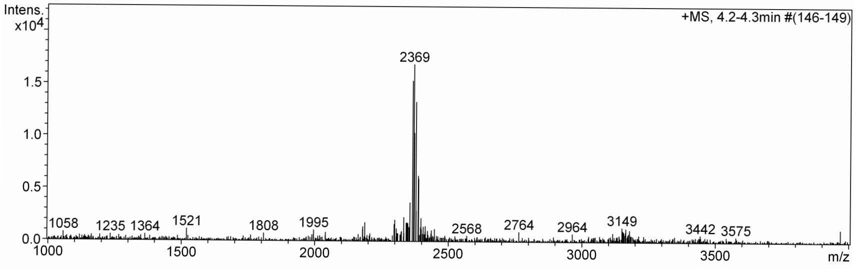

Bruker Daltonics DataAnalysis 3.1 printed: 16/07/2013 15:07:28 Page 1 of 1

```
Data File C:\HPCHEM\1\DATA\GMILLER4\SU 1491 3CM COL 16TH JULY.D
 Sample Name: SU 1491 3cm col 16th July
   ===================================================================
   Acq. Operator    : Rehana                            Seq. Line :   1
   Acq. Instrument  : Instrument 1                       Location : Vial 1
   Injection Date   : 16/07/2013 13:04:40                     Inj :   1
                                                      Inj Volume : 20 µl
   Different Inj Volume from Sequence !     Actual Inj Volume : 5 µl
   Sequence File    : C:\HPCHEM\1\SEQUENCE\GAVIN MILLER.S
   Acq. Method      : C:\HPCHEM\1\METHODS\GAVIN MILLER 3CM COL 100ACN.M
   Last changed     : 16/07/2013 13:03:25  by Rehana
   Analysis Method  : C:\HPCHEM\1\METHODS\GAVIN MILLER 3CM COL 100ACN.M
   Last changed     : 16/07/2013 14:48:02  by Rehana
   Sample Info      : Gavin Miller                     SU 1491       16th July

                      Supelco C8 4.6x30mm 5u   100% ACN  5ul inj
                      ESI source  +ve ES 230nm 0.5ml/min
```

===

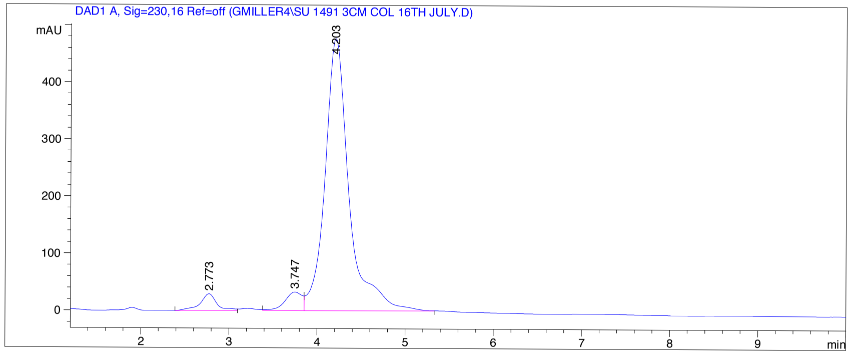


```
===================================================================
                         Area Percent Report
===================================================================

Sorted By             :      Signal
Multiplier            :      1.0000
Dilution              :      1.0000
Use Multiplier & Dilution Factor with ISTDs


Signal 1: DAD1 A, Sig=230,16 Ref=off

Peak RetTime Type  Width     Area      Height     Area
  #   [min]        [min]   [mAU*s]     [mAU]        %
----|-------|----|-------|----------|----------|--------|
   1   2.773 BV    0.1920  388.38840   29.42808   3.9273
   2   3.747 VV    0.2029  445.82135   32.73233   4.5080
   3   4.203 VB    0.2795 9055.34082  476.96234  91.5647

Totals :                   9889.55057  539.12276

===================================================================
                          *** End of Report ***
```

Instrument 1 16/07/2013 14:48:07 Rehana Page 1 of 1

Supplementary Figure 7: MALDI TOF MS and Isotope Pattern for **S1**

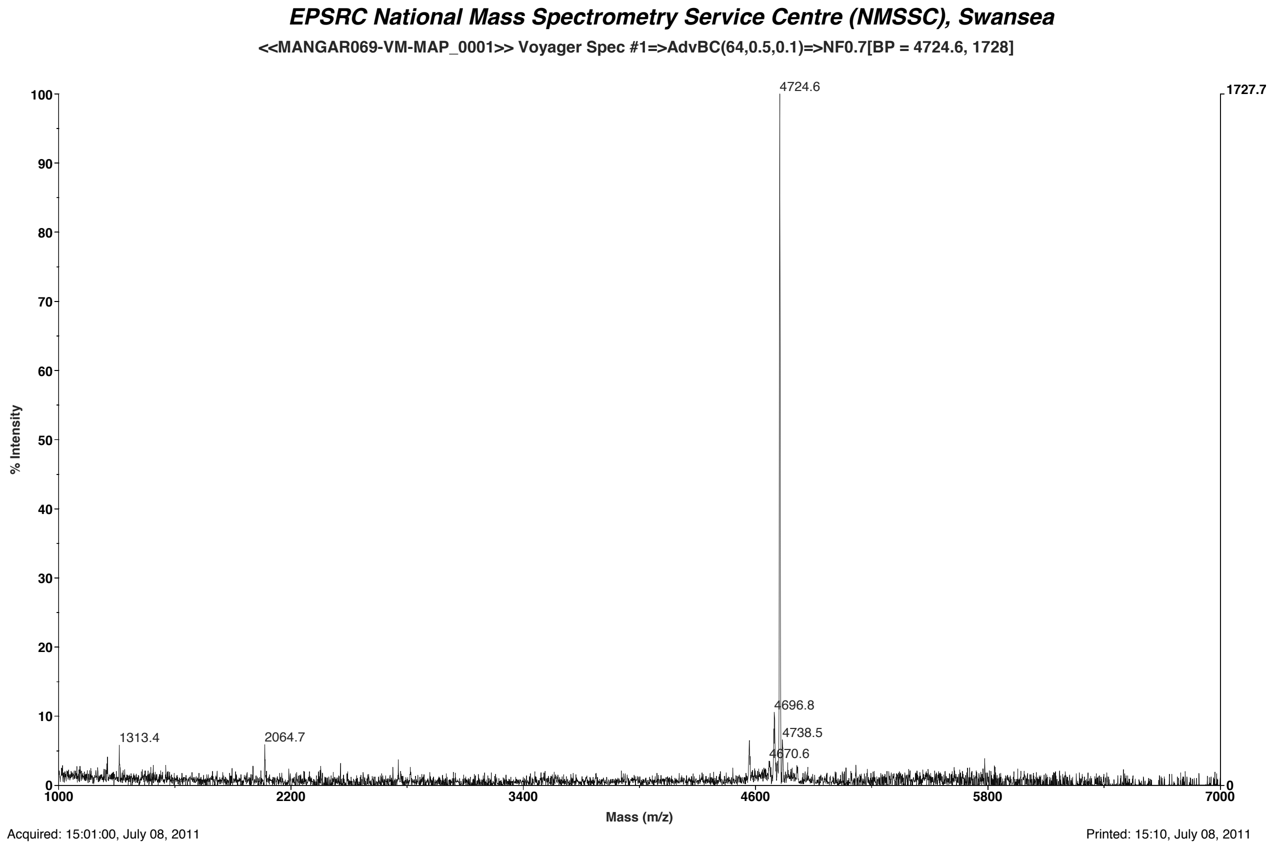

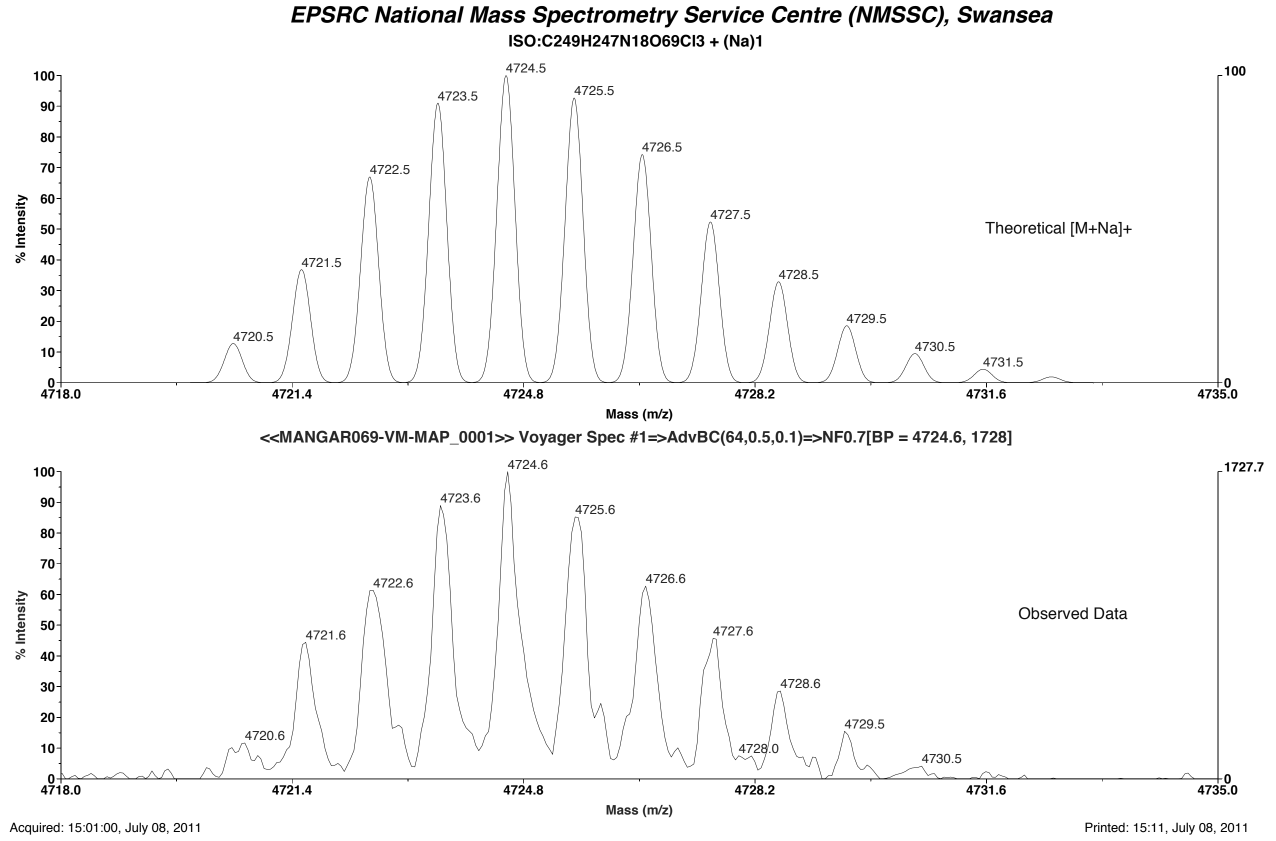

Supplementary Figure 8: ^{1}H NMR (400 MHz; CD_3OD) spectrum for **6**

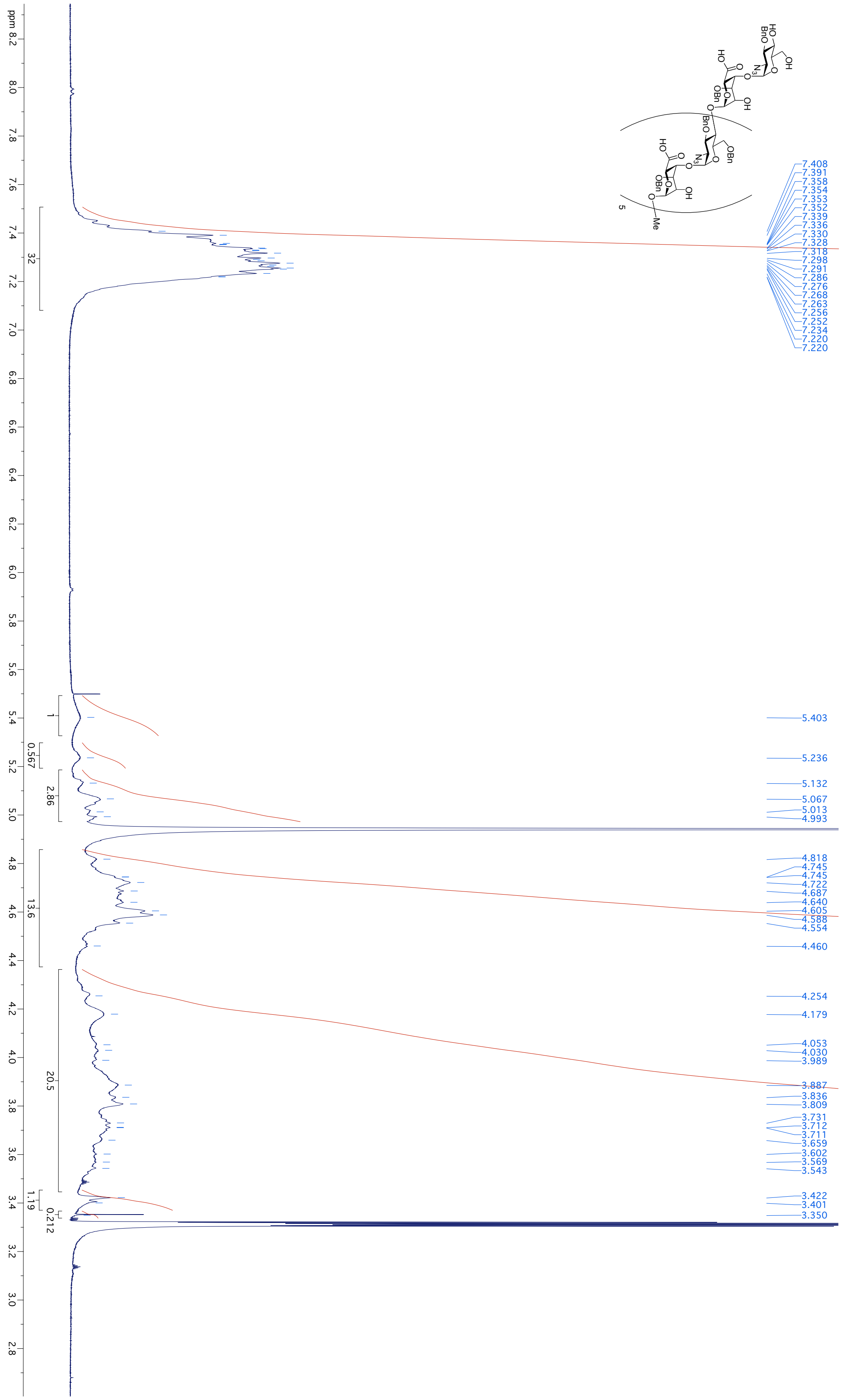

Supplementary Figure 9: COSY NMR (400 MHz; CD_3OD) spectrum for **6**

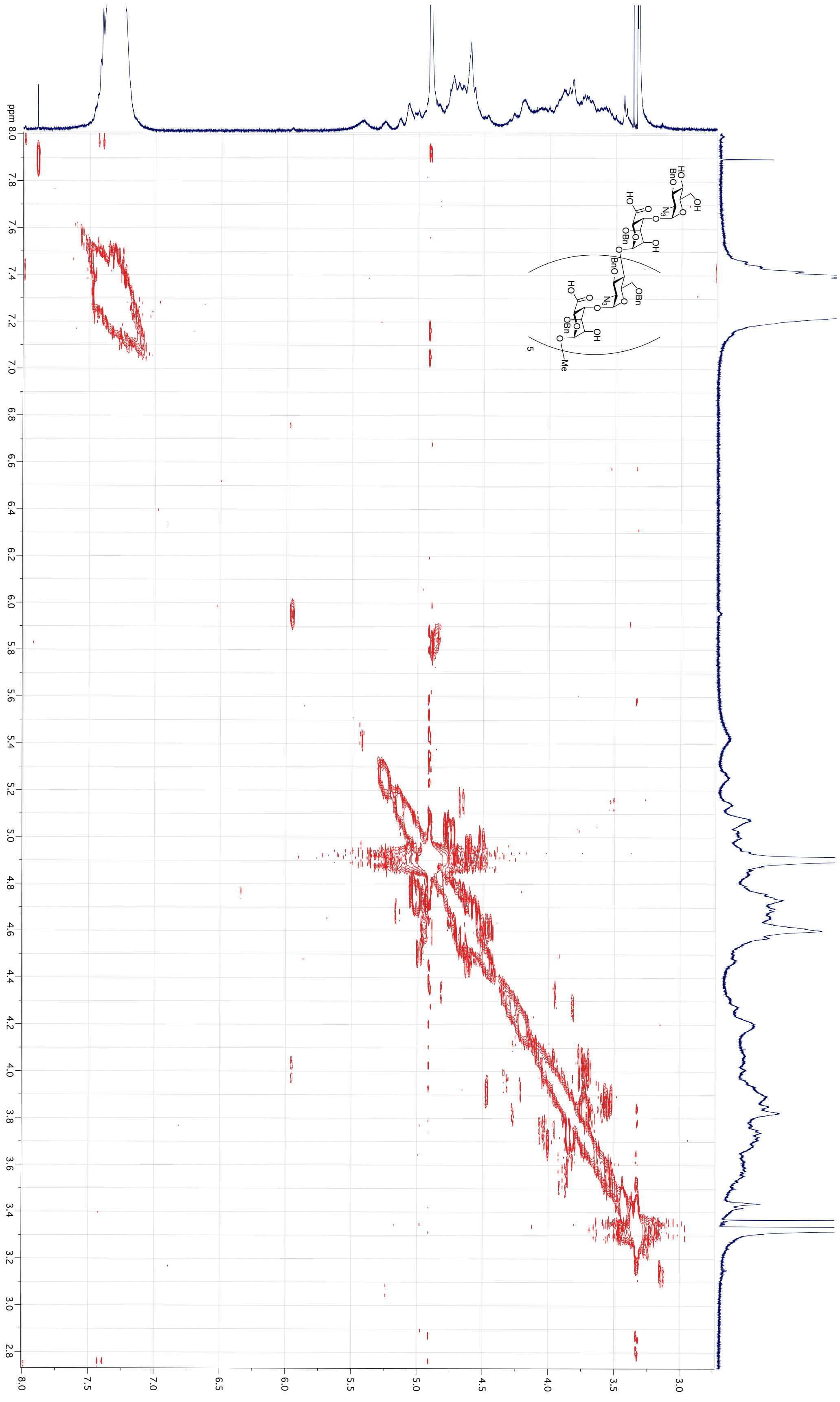

Supplementary Figure 10: FT MS spectrum and isotope pattern for **6**

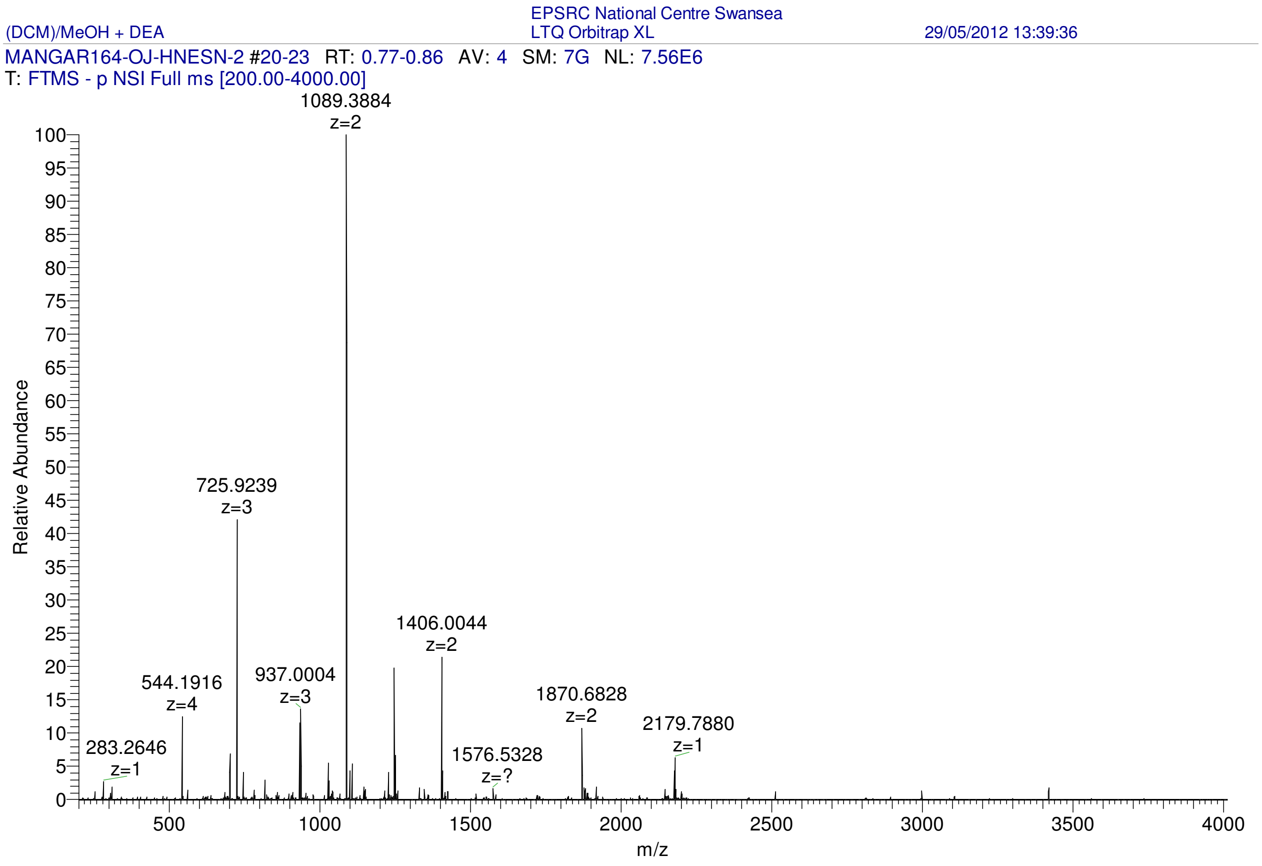

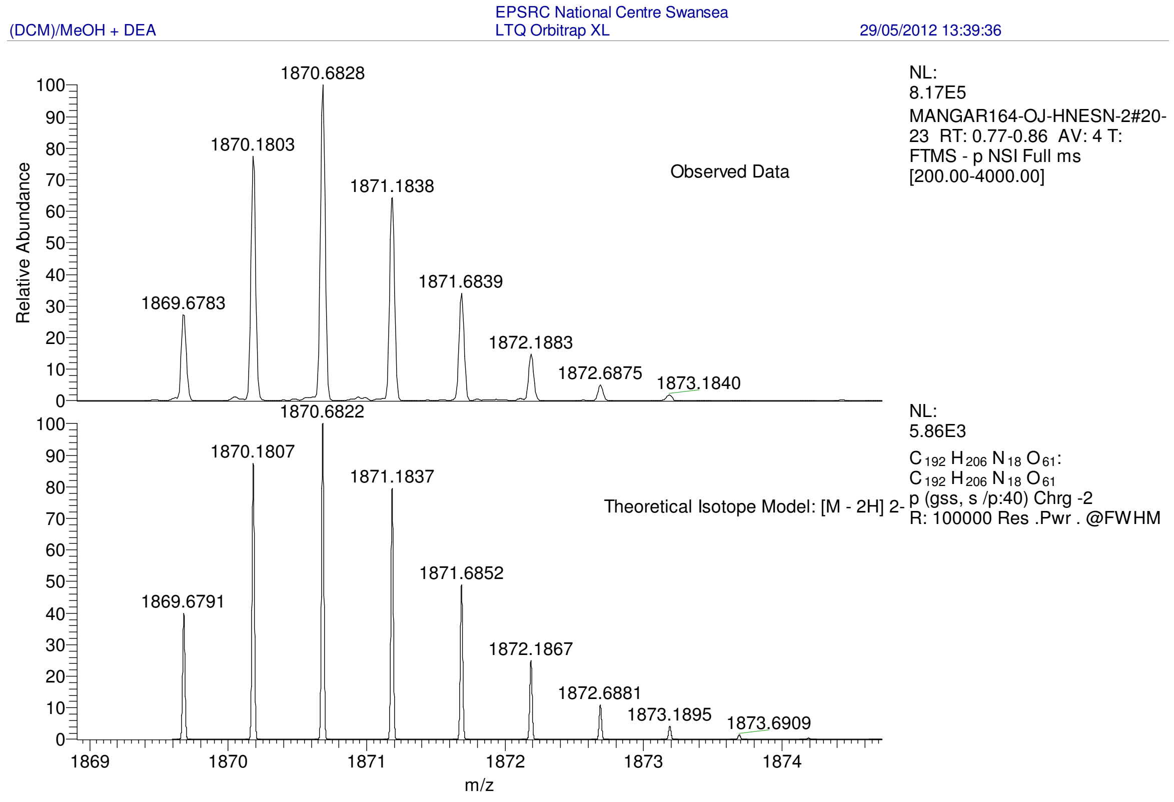

Supplementary Figure 11: ^{1}H NMR (400 MHz; CD_3OD) spectrum for **7**

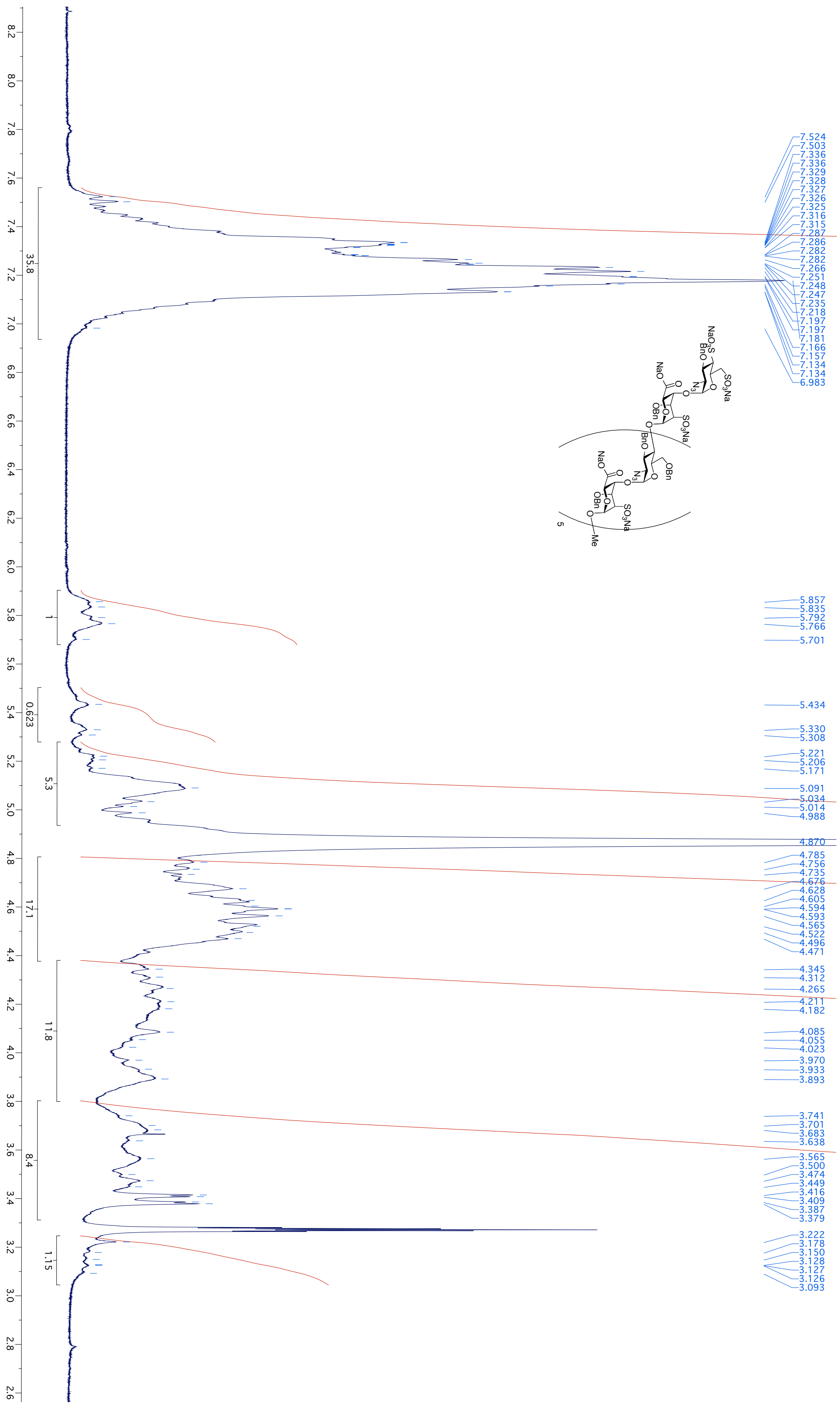

Supplementary Figure 12: COSY NMR (400 MHz; CD_3OD) spectrum for **7**

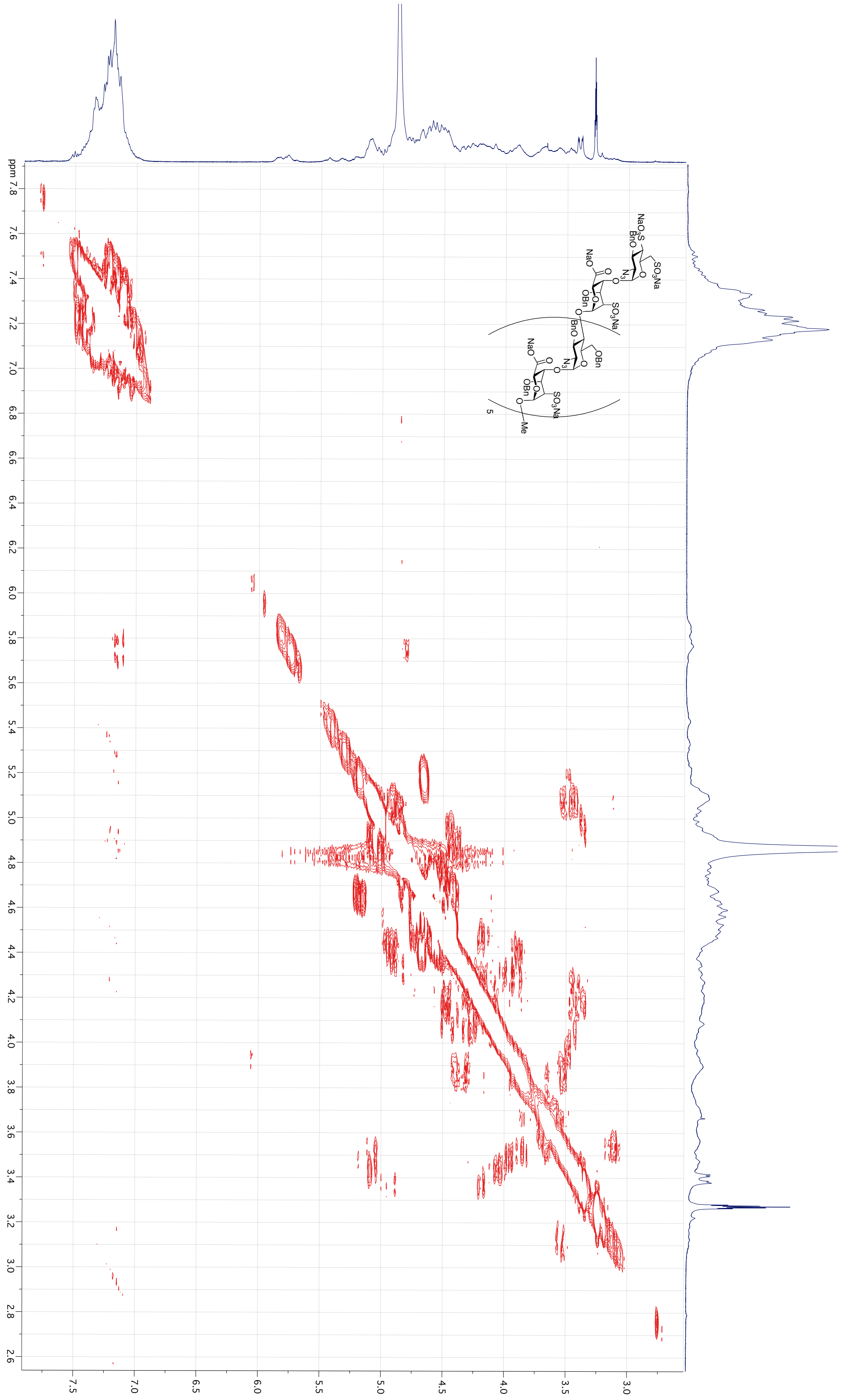

Supplementary Figure 13: FT MS for **7**

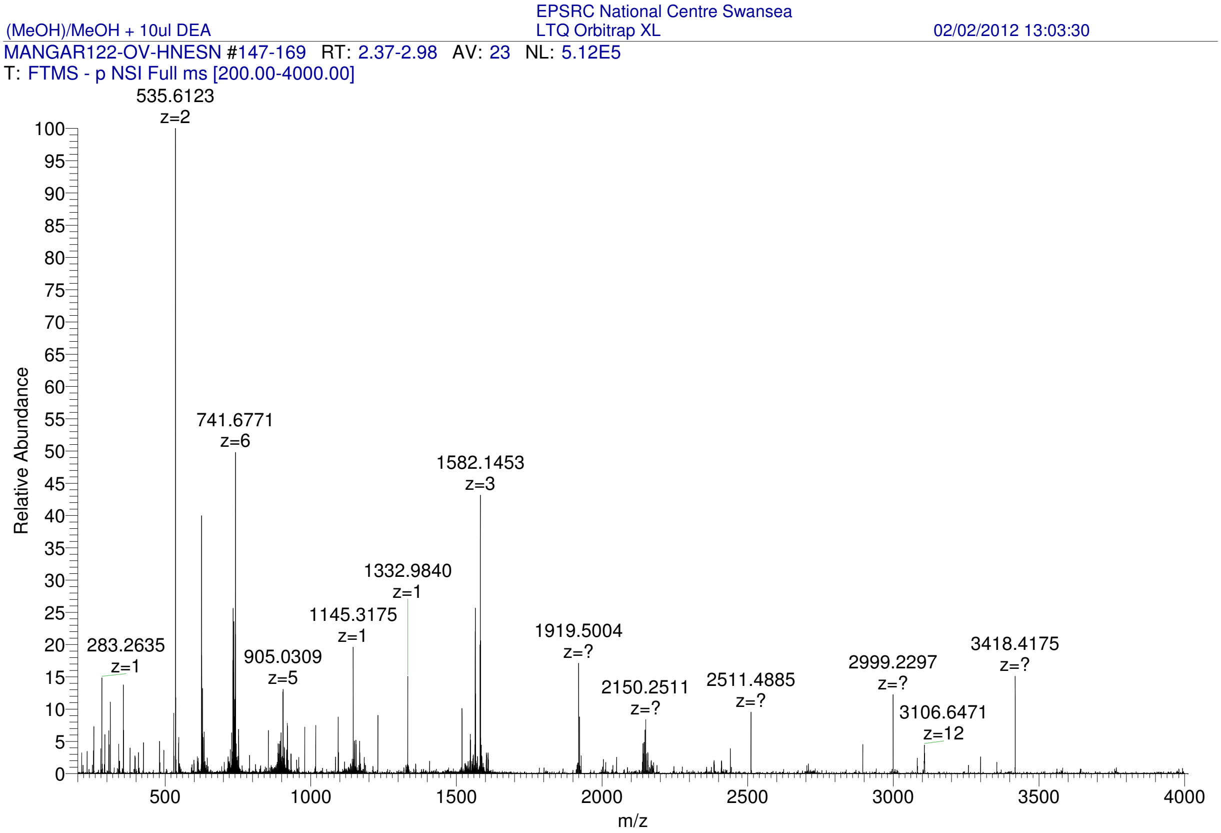

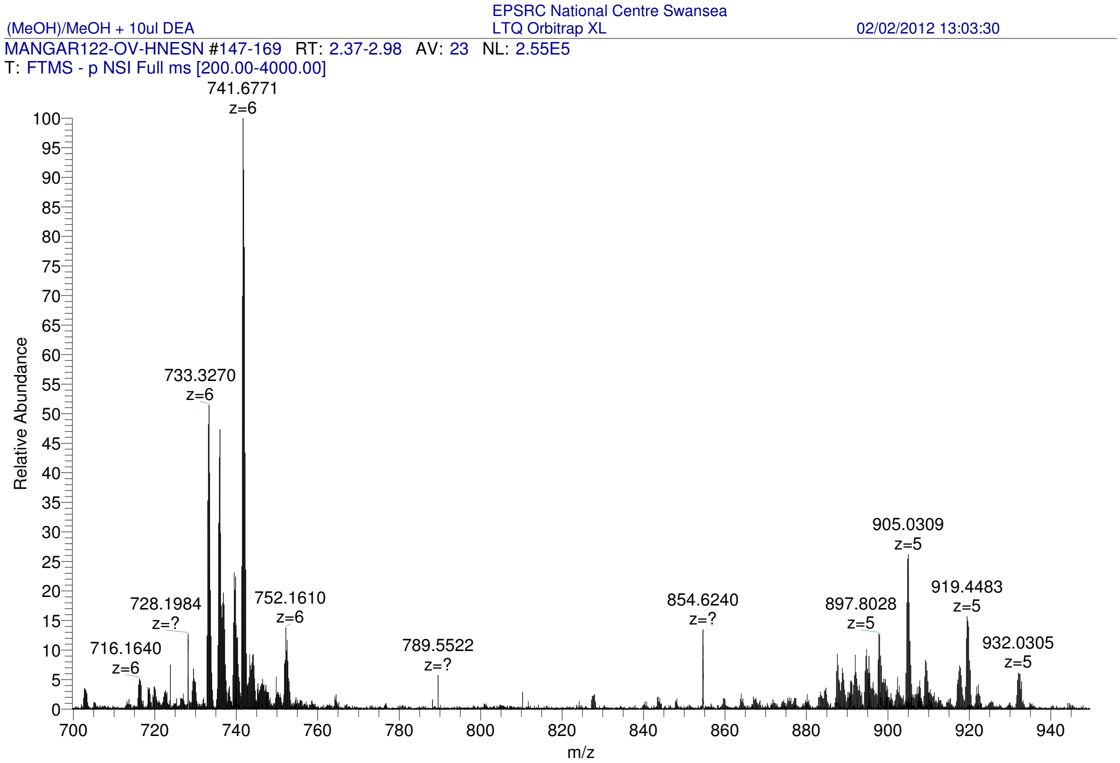

Supplementary Figure 14: ^{1}H NMR (400 MHz; D_2O) spectrum for **8**

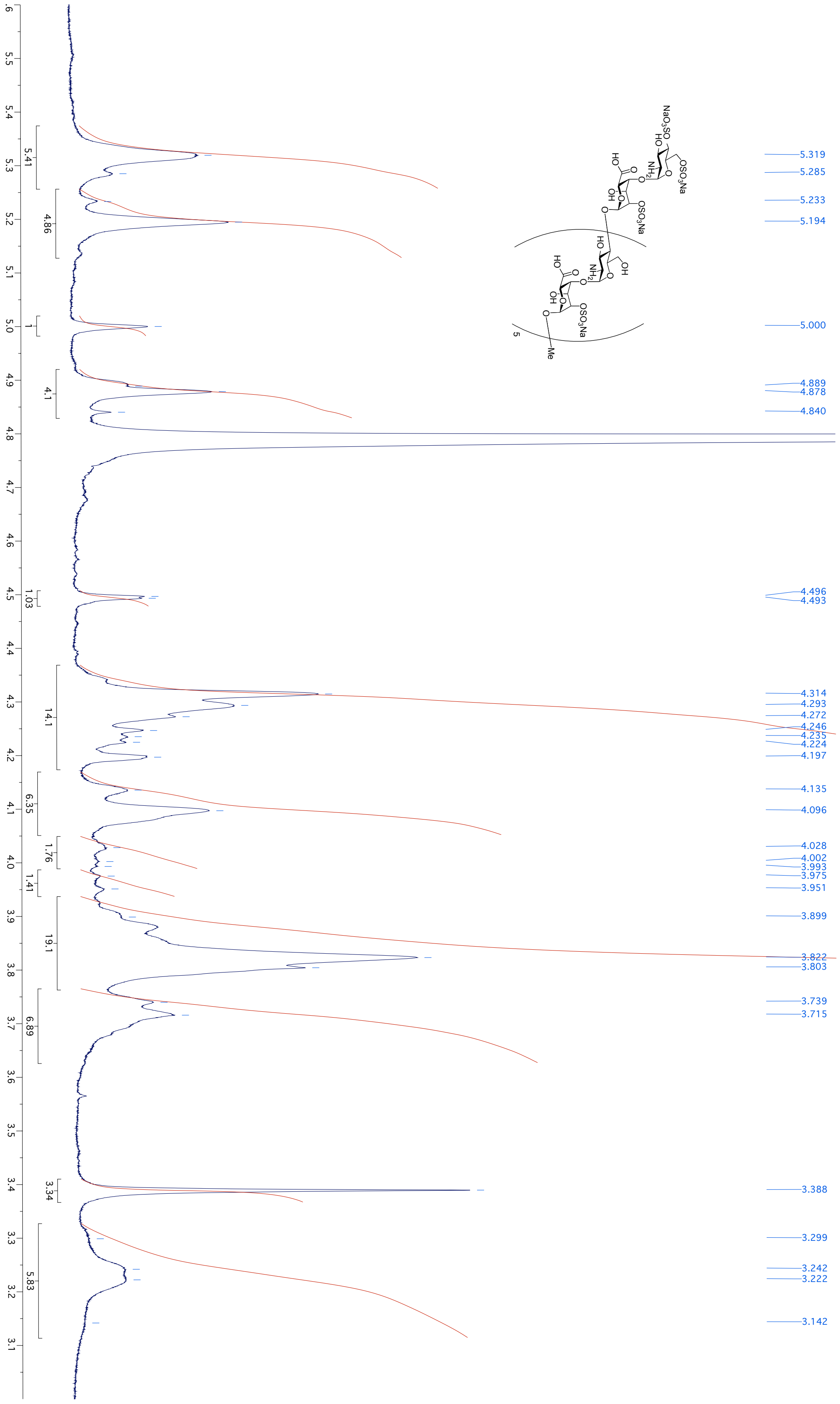

Supplementary Figure 15: COSY NMR (400 MHz; D_2O) spectrum for **8**

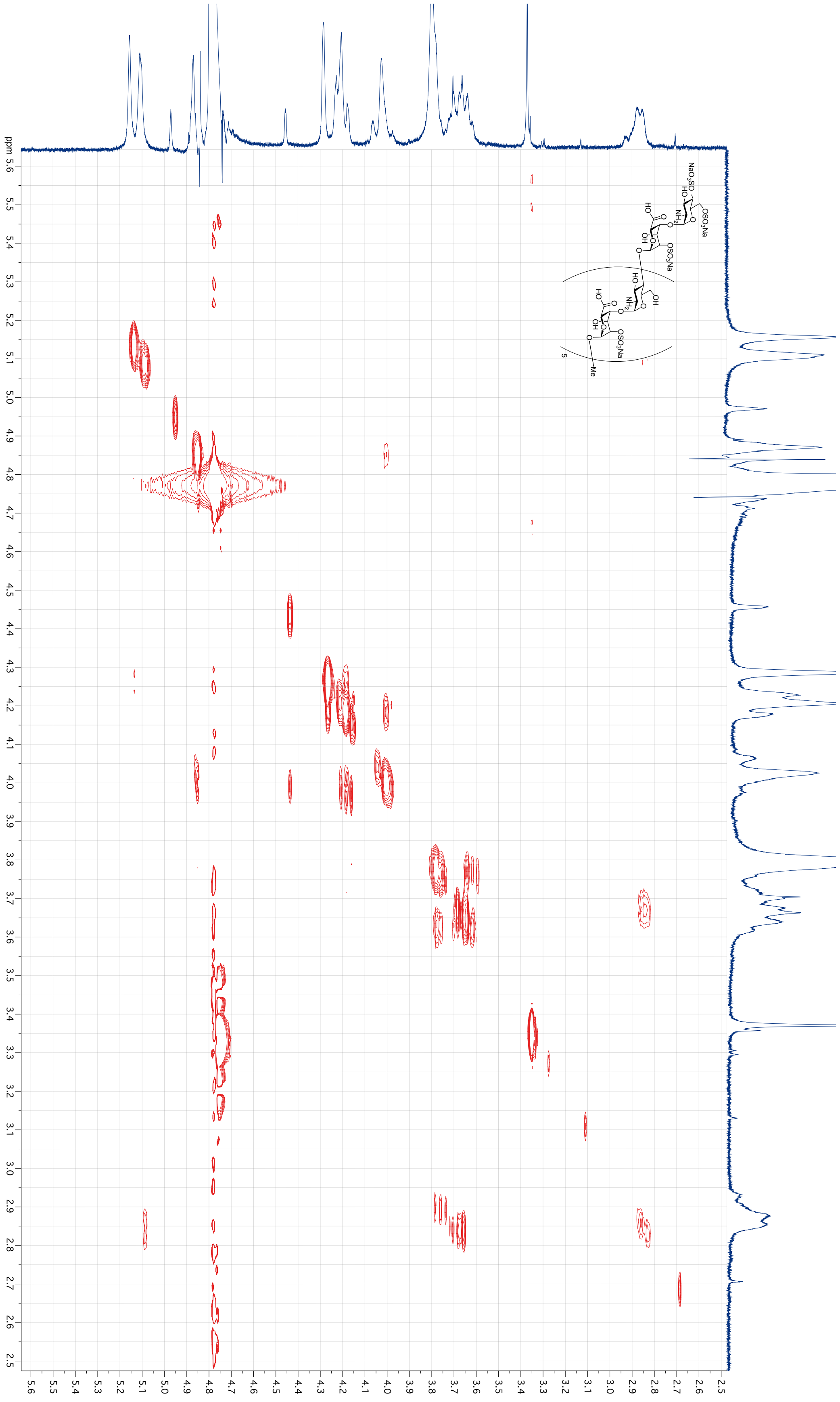

Supplementary Figure 16: FT MS spectrum and isotope pattern for **8**

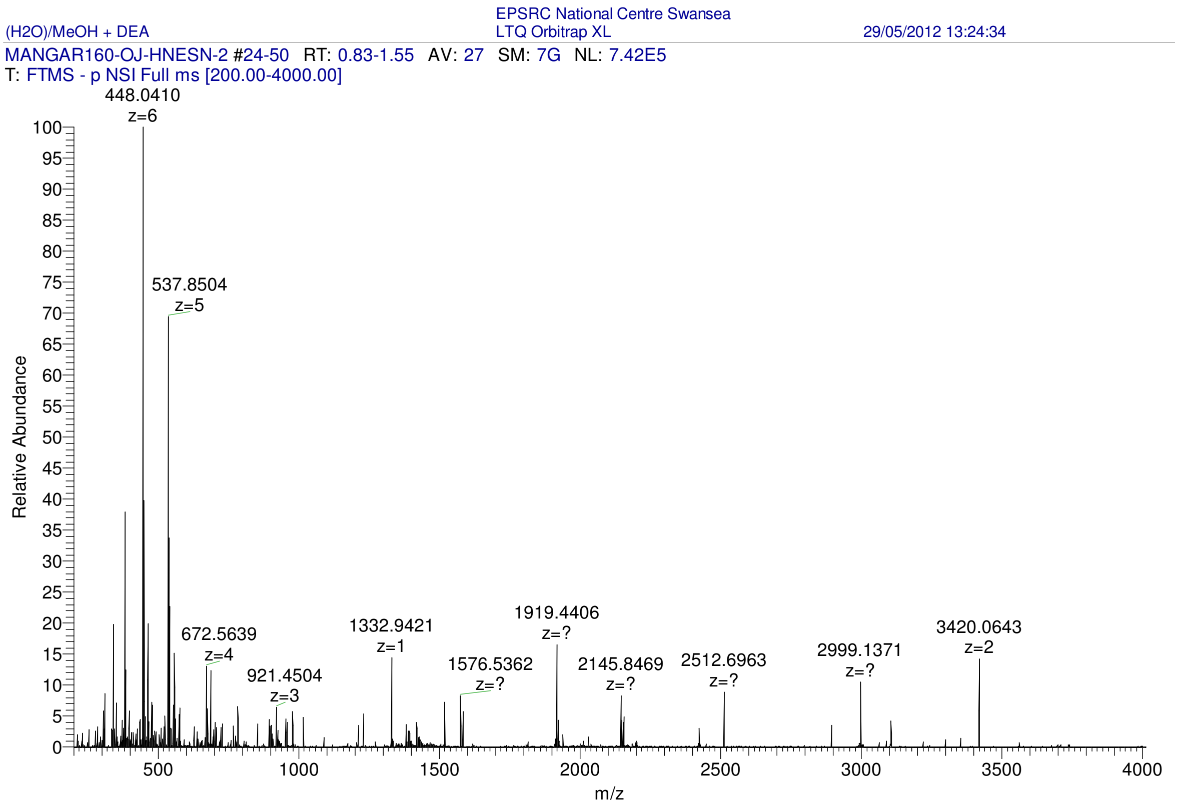

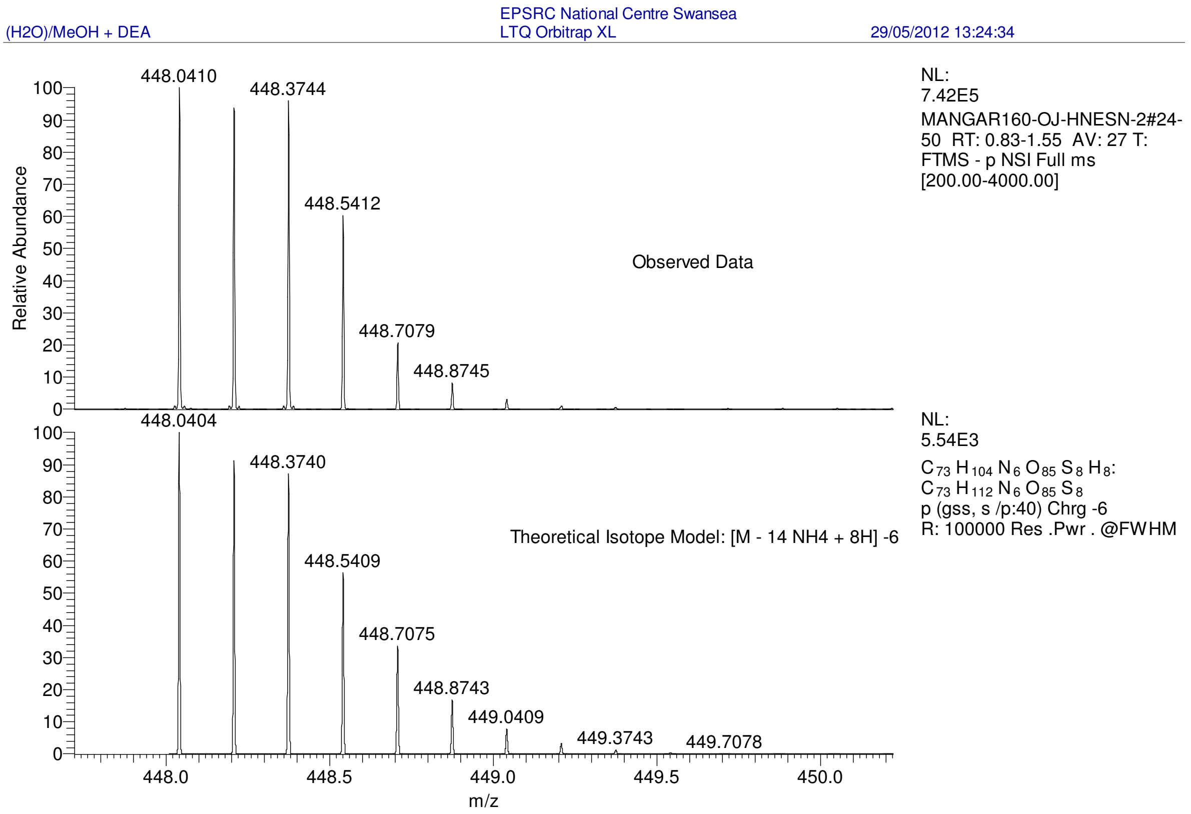

Supplementary Figure 17: ^{1}H NMR (400 MHz; D_2O) spectrum for **2**

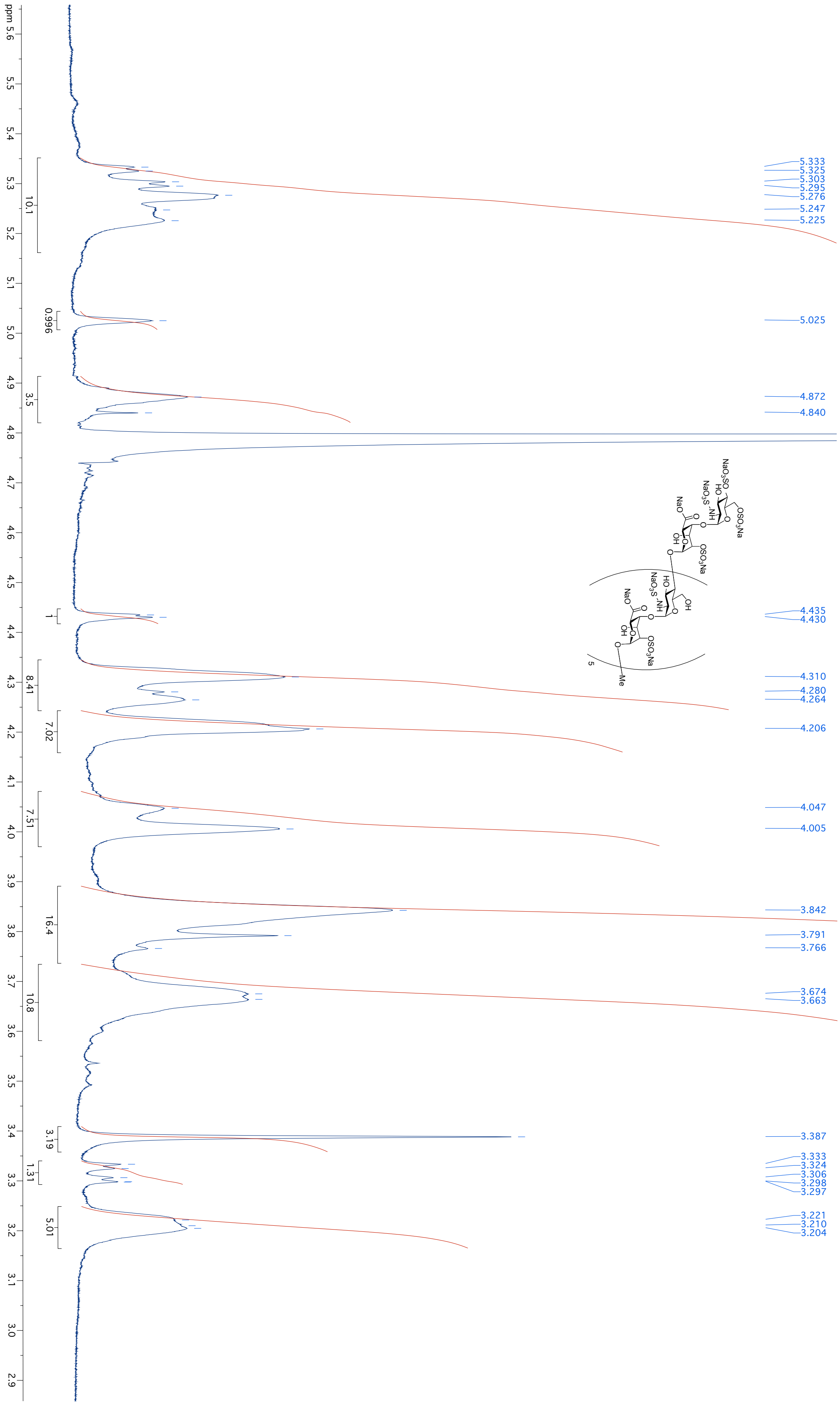

Supplementary Figure 18: ^{1}H NMR (800 MHz; D_2O) spectrum for **2**

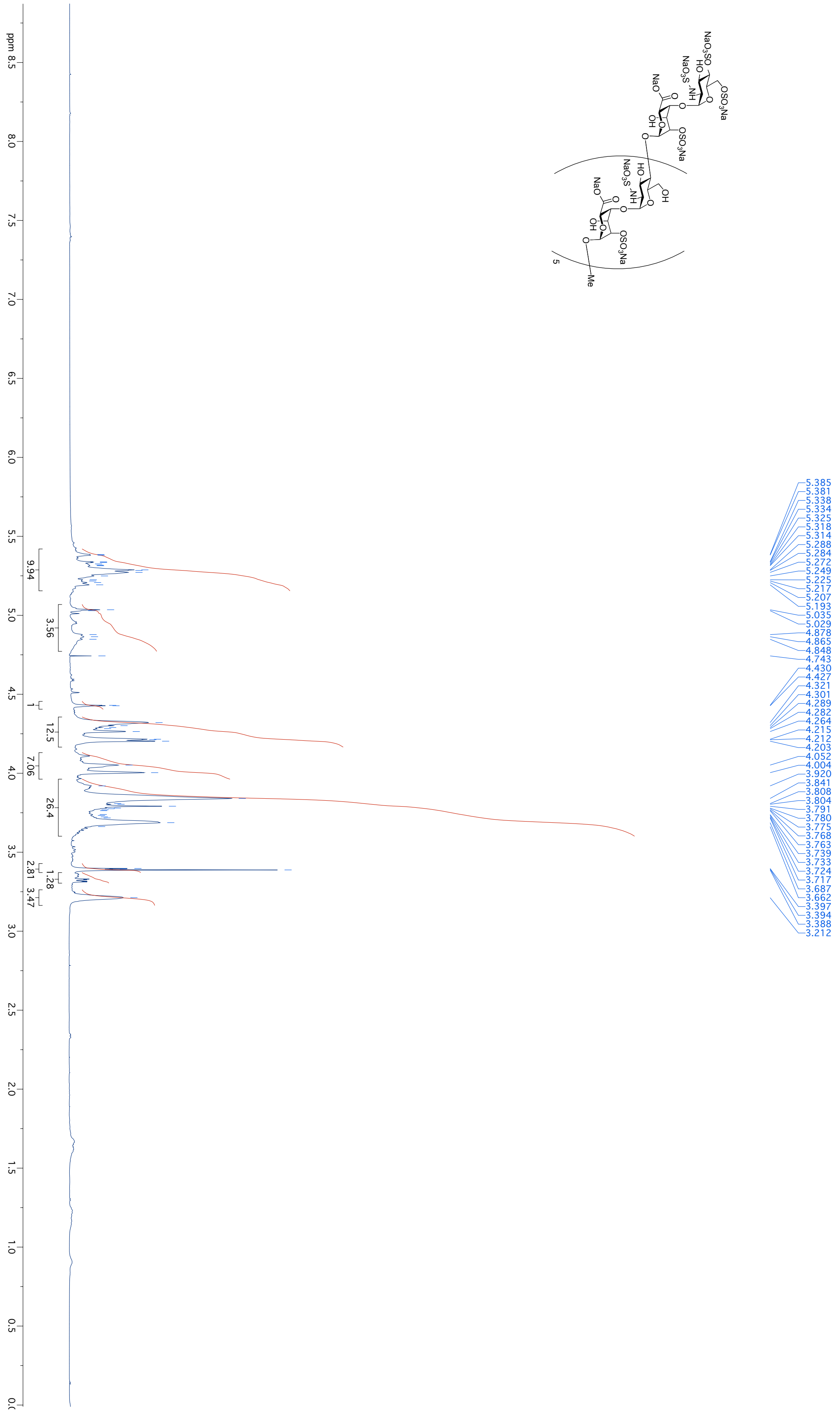

Supplementary Figure 19: HSQC NMR (800 MHz; D_2O) spectrum for **2**

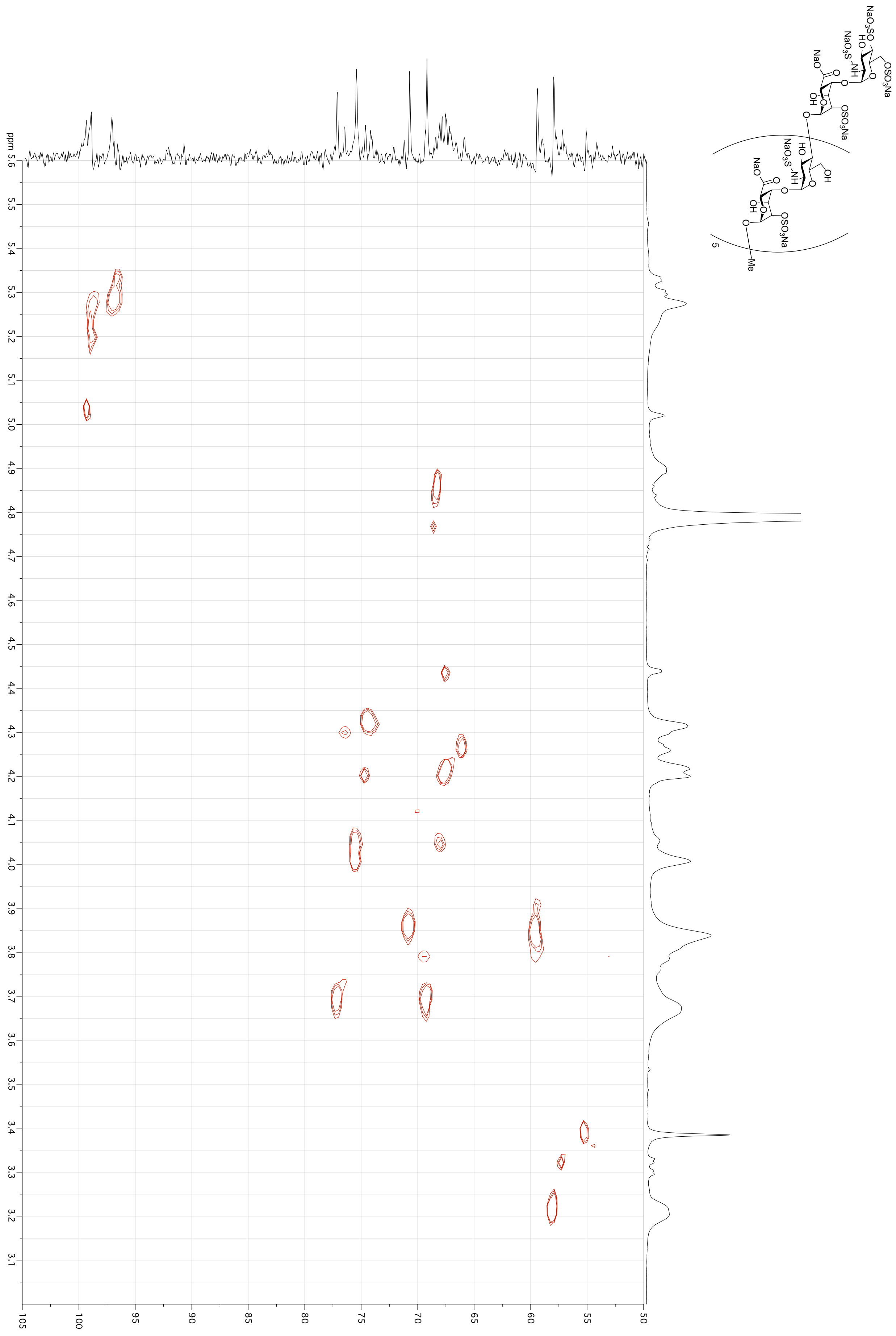

Supplementary Figure 20: ^{13}C NMR (200 MHz; D_2O) spectrum for **2**

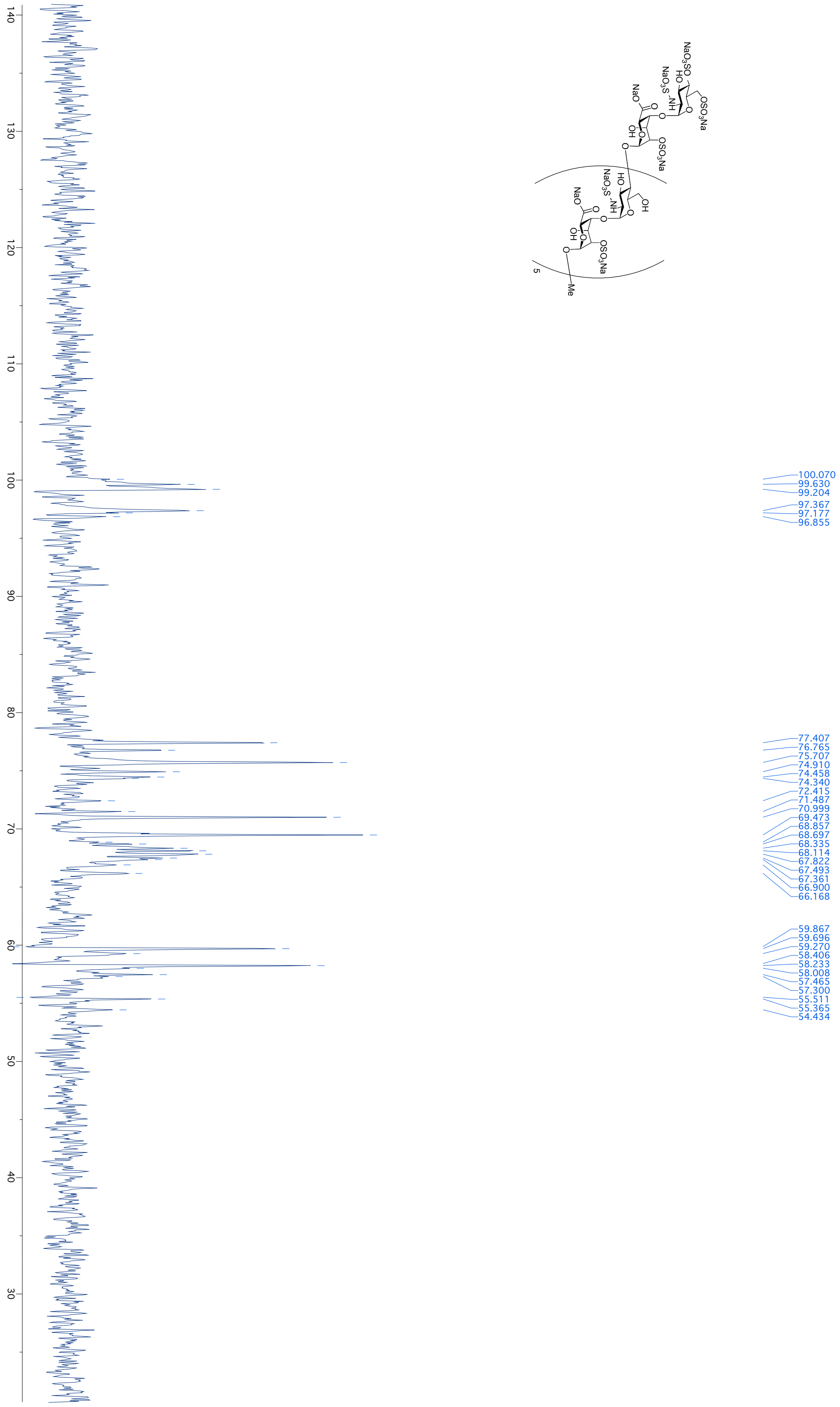

Supplementary Figure 21: FT MS spectrum for **2**

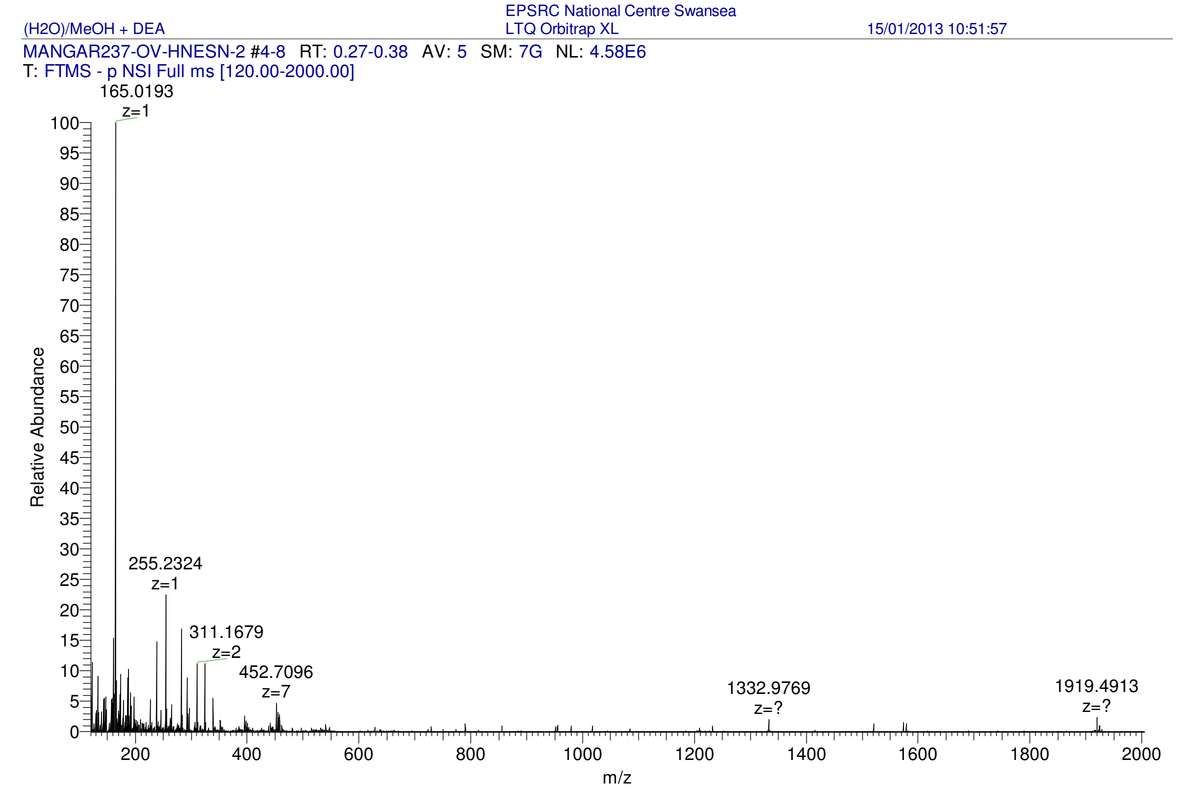

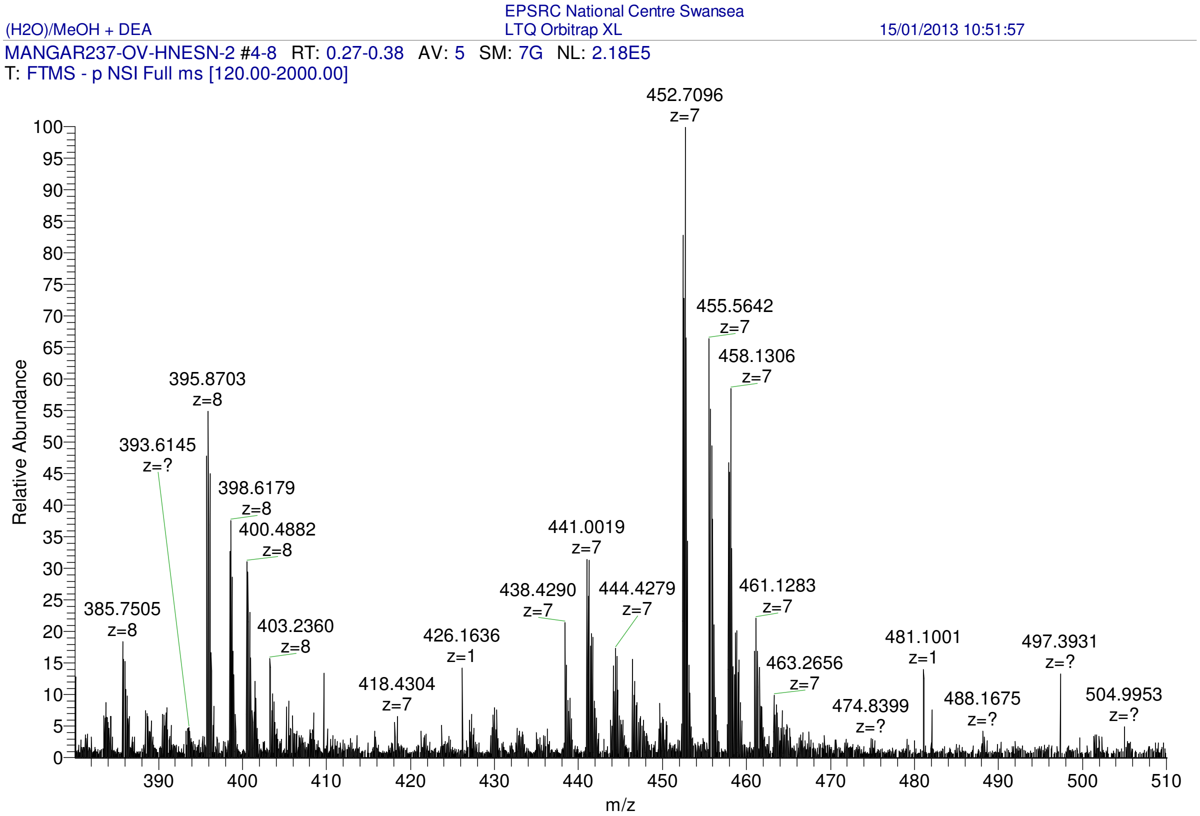

Supplementary Figure 22: HSQC NMR (800 MHz; D_2O) overlay for **2** and **1** (crosspeaks for **2** are blue, **1** are red)

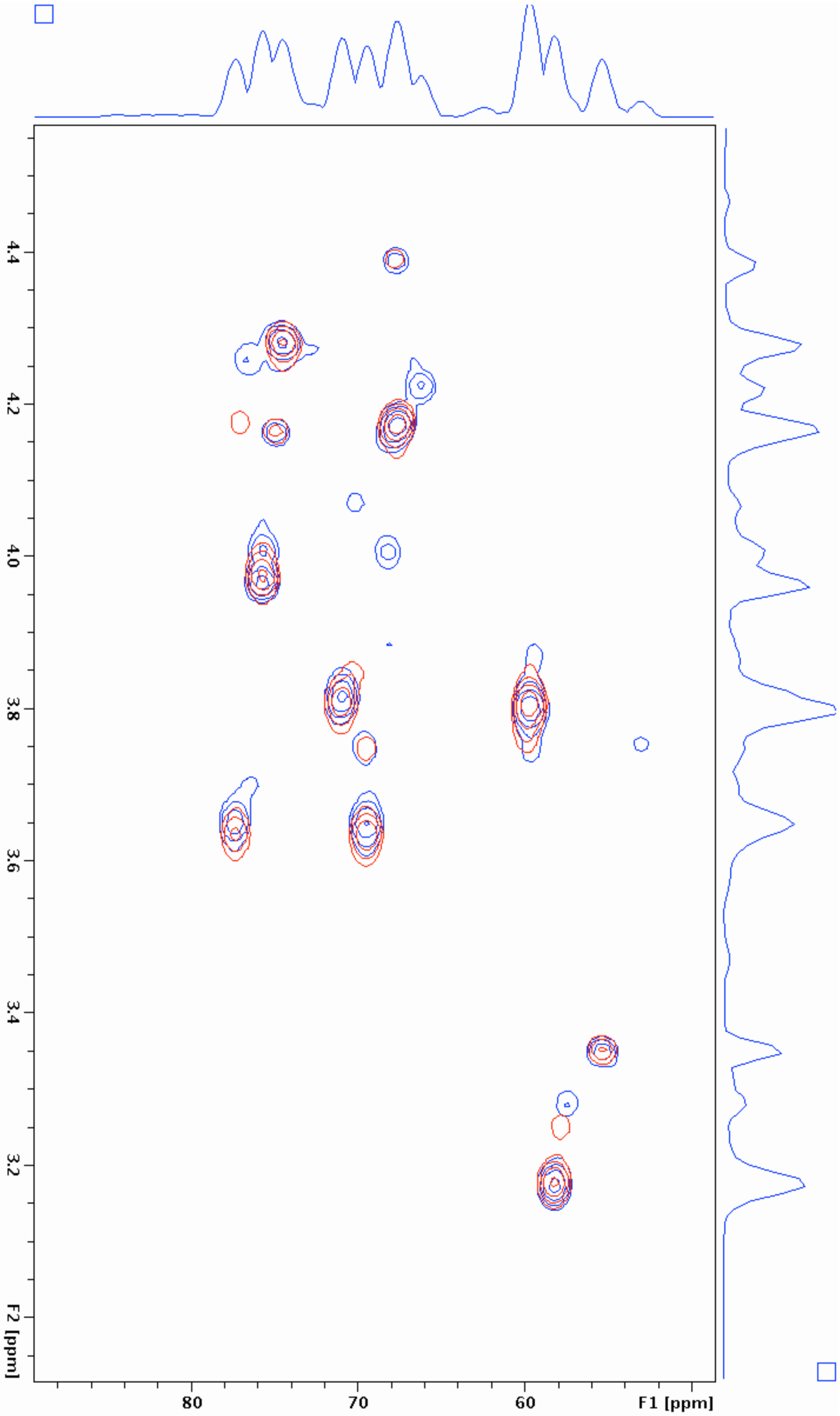

Biology Figures

Supplementary Figure 23. Effect of oligosaccharides on CXCL12- and CXCL8-induced signalling. (a) 12-mer ISNSm6S showed the greatest potency in inhibiting CXCL12-induced ERK phosphorylation. Serum-starved HUVECs were stimulated with 50 ng/mL of CXCL12 for 10 minutes in the absence or presence of oligosaccharides. Stimulation by CXCL12 alone was taken as 100%. Average values derived from four independent experiments are expressed as the mean ±SEM. Average values for treatment with a heparin 12-mer were derived from two independent experiments and expressed as the mean ±SD. (**b**) oligosaccharide ISNS is a more potent inhibitor of CXCL8 than CXCL12. IL-8 (50 ng/mL) stimulation of serum-starved HUVECs was performed for 10 minutes in the absence or presence of respective oligosaccharides. Phosphorylation of STAT3 was detected with the antibody recognizing phosphorylated serine 727. Stimulation with CXCL8 alone was defined as 100%. Average values derived from densitometric evaluation of four independent experiments were expressed as the mean ±SEM. The average effect of a heparin 12-mer was derived from two independent experiments and expressed as the mean ±SD.

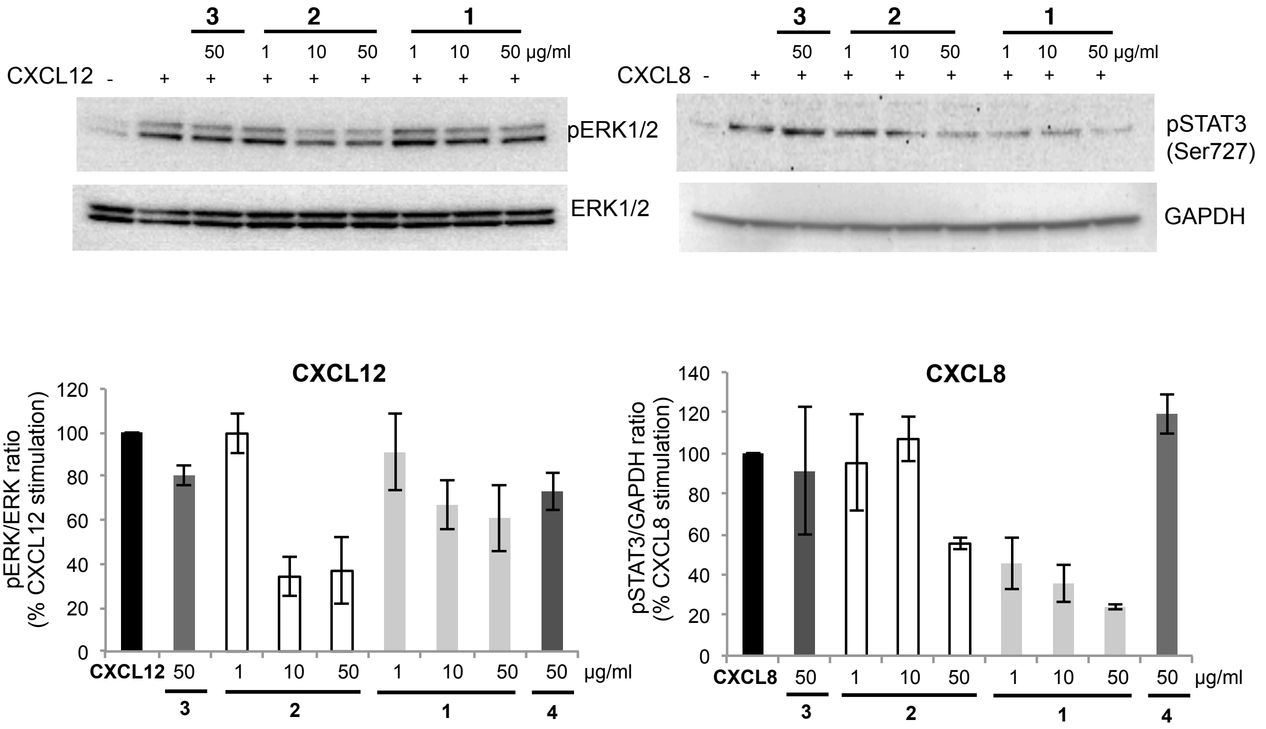

Supplementary Figure 24: Specificity of oligosaccharides in inhibiting cytokine-induced neo-vascularization and neutrophil and macrophage infiltration into *in vivo* implanted sponges. (**a-b**) oligosaccharide **1** shows specificity in inhibiting neo-vascularization (**a**) and neutrophil infiltration (**b**) into CXCL8-impregnated agarose-Gelfoam sponges. (**a**) Blood vessels and their coverage with mural cells were visualised by immunostaining of sponge sections with the antibodies against mouse CD31 (green) and α-SMA (red), respectively. Nuclei were visualized by staining with Hoechst. Scale bars, 200 µm. (**b**) Neutrophils were detected by staining with Ly-6B.2 neutrophil binding antibody (green). Nuclei were stained with Hoechst (blue). Scale bars represent 200 µm. (**c-e**) 12-mer **2** targets CXCL12-induced angiogenesis and neutrophil and macrophage infiltration into sponges. (**c**) Sections from CXCL12-impregnated sponges with or without oligosaccharides were co-stained with anti-CD31 (green) and anti-α-SMA (red) antibodies to visualize blood vessel infiltration and their coverage with mural cells. Scale bars represent 200 µm. (**d**) Neutrophil infiltration was detected by staining with anti-Ly-6B.2 antibody (green). Hoechst staining was used to visualize nuclei (blue). Scale bars, 200 µm. (**e**) Macrophages were visualised by immunostaining of sponge sections with anti-F4/80 antibody which recognizes specific macrophage F4/80 receptor (green), while nuclei were visualized by Hoechst staining. Scale bars, 200 µm.

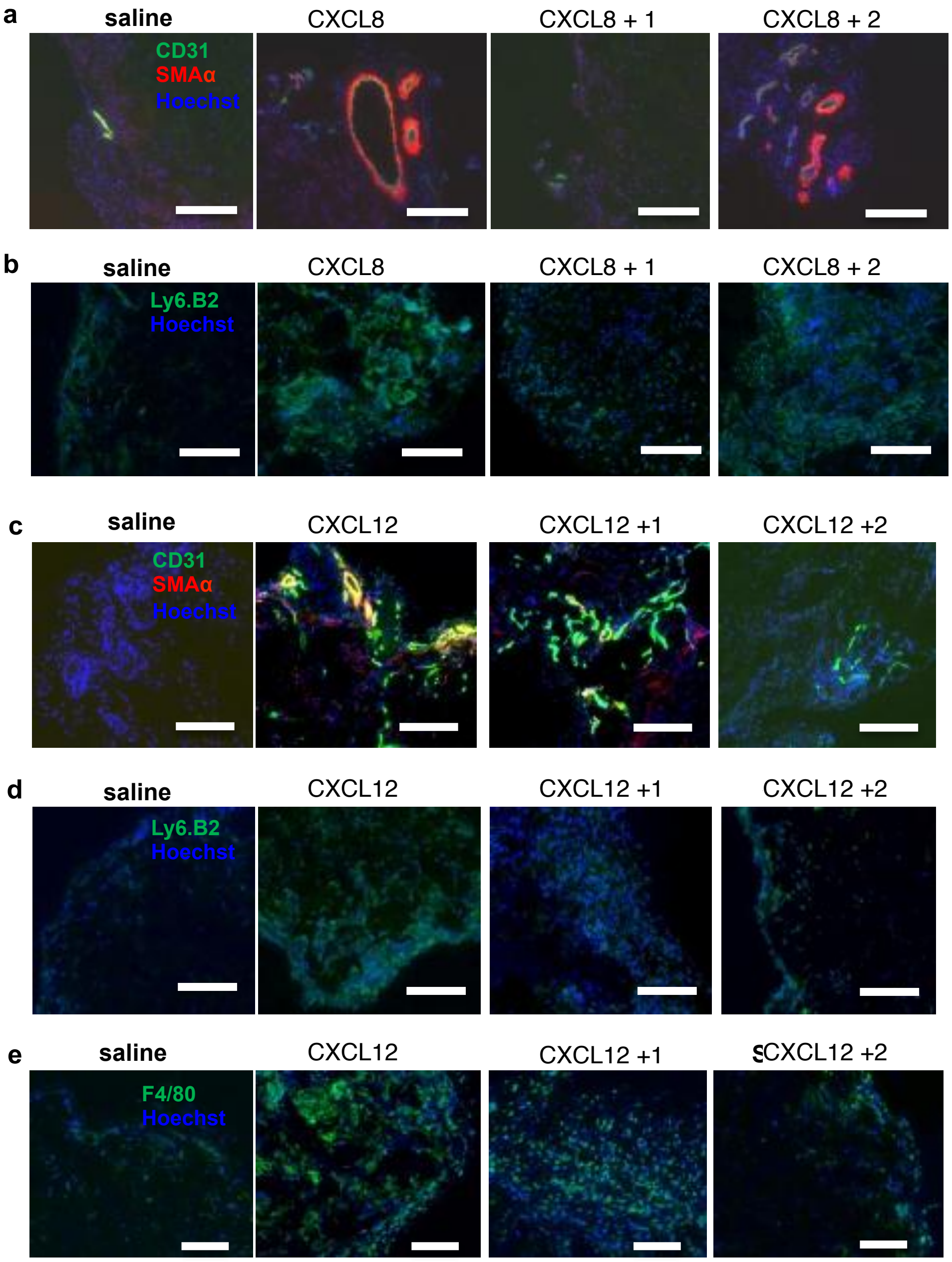

Supplementary Figure 25: Comparison of the effects of heparin-derived dodecasaccharide and oligosaccharide 3 on endothelial cell migration. The effects of dodecasaccharides on CXCL-8 and CXCL12-induced HUVEC migration were tested in a wound healing assay. The impact of cytokines on wound healing without oligosaccharides is expressed as 100%. Oligosaccharides **3** and **dp12** were added at 50 µg/ml. The values are derived from two independent experiments performed in triplicate and represent the mean ± SD. *, p 0.006; ‡, p 0.05**.**

dp12 contains a terminal C=C in place of the polar 4-OH group. The synthetic 4-OS systems reported here retain the terminal ring conformation but the close comparability of dp12 and synthetic per-O-6 sulfated 3 confirms that the 4-OS is not a significant variable in any of the effects seen here.

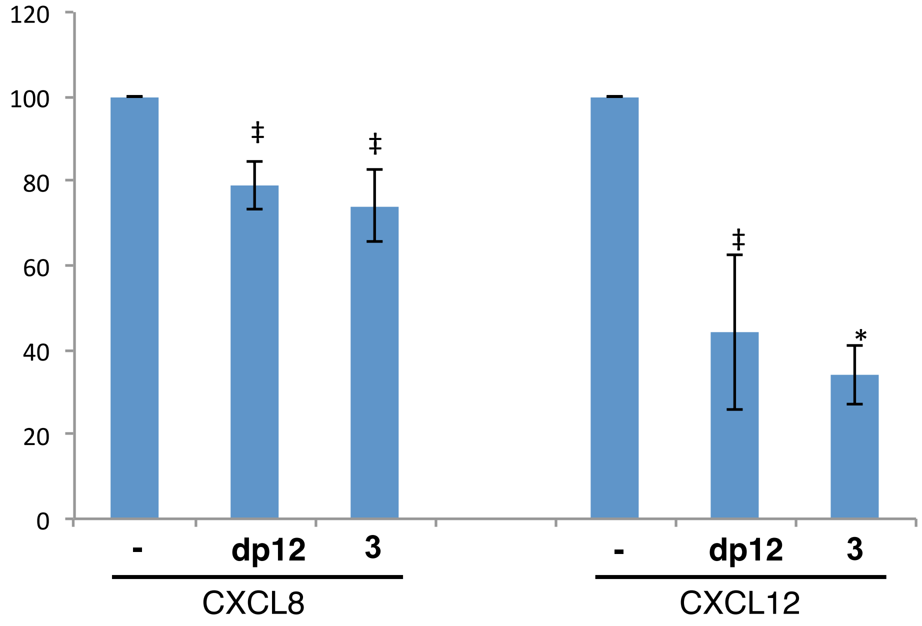

Supplementary Figure 26: Statistical data for Fig.2c and additional data covering oligosaccharide 3.

Table 1. P-values generated by comparing the means of CXCL12 and CXCL8 binding to HS without oligosaccharides (controls) and in the presence of 100 µg/ml of each oligosaccharide.

	100 µg/ml **2** vs control (no oligo)	100 µg/ml **1** vs control (no oligo)	100 µg/ml **3** vs control (no oligo)	100 µg/ml heparin dp12 vs control (no oligo)
CXCL8	0.01	0.0017	0.0008	0.0005
CXCL12	0.05	0.0002	0.067	0.0028

Table 2. P-values generated for the inhibition of IL-8 and SDF-1α binding to HS by ISNS and ISNSmono6S oligosaccharides.

	0.1 µg/ml **2** vs 0.1 µg/ml **1**	1 µg/ml **2** vs 1µg/ml **1**	10 µg/ml **2** vs 10 µg/ml **1**	100 µg/ml **2** vs 100 µg/ml **1**
CXCL8	0.0025	$3x10^{-5}$	0.0017	0.027
CXCL12	0.041	0.265	0.023	$3.6x10^{-5}$

Table 3. P-values generated by comparing the mean values of IL-8 and SDF-1α binding to HS in the presence of ISNS6S oligosaccharide with the values of ISNS or mono6S oligosaccharide treatment.

	0.1 µg/ml **3** vs 0.1 µg/ml **2**	1 µg/ml **3** vs 1 µg/ml **2**	10 µg/ml **3** vs 10 µg/ml **2**	100 µg/ml **3** vs 100 µg/ml **2**	0.1 µg/ml **3** vs 0.1 µg/ml **1**	1 µg/ml **3** vs 1 µg/ml **1**	10 µg/ml **3** vs 10 µg/ml **1**	100 µg/ml **3** vs 100 µg/ml **1**
CXCL8	0.47	0.00367	$2x10^{-5}$	0.977	$1.4x10^{-5}$	0.00018	0.0058	0.071
CXCL12	0.14	0.407	0.105	0.42	0.068	0.0024	0.184	0.367

Table 4. P-values generated for the inhibition of IL-8 and SDF-1α binding to HS by heparin dp12 and ISNS or ISNSmono6S oligosaccharides.

	0.1 µg/ml **1** vs 0.1 µg/ml heparin dp12	1 µg **1** vs 1 µg/ml heparin dp12	10 µg/ml **1** vs 10 µg/ml heparin dp12	100 µg/ml **1** vs 100 µg/ml heparin dp12	0.1 µg/ml **2** vs 0.1 µg/ml heparin dp12	1 µg/ml **2** vs 1 µg/ml heparin dp12	10 µg/ml **2** vs 10 µg/ml heparin dp12	100 µg/ml **2**vs 100 µg/ml heparin dp12
CXCL8	0.992	0.0004	0.021	0.0048	0.58	0.142	0.00049	0.658
CXCL12	0.05	0.97	0.26	0.012	0.079	0.03	0.118	0.0005
